# Carbon Dots Based Photoinduced Reactions: Advances and Perspective

**DOI:** 10.1002/advs.202207621

**Published:** 2023-02-03

**Authors:** Yue Yu, Qingsen Zeng, Songyuan Tao, Chunlei Xia, Chongming Liu, Pengyuan Liu, Bai Yang

**Affiliations:** ^1^ State Key Laboratory of Supramolecular Structure and Materials College of Chemistry Jilin University Changchun 130012 P. R. China; ^2^ Department of Materials Science and Engineering Seoul National University 1 Gwanak‐ro, Gwanak‐gu Seoul 08826 Republic of Korea

**Keywords:** carbon dots, carbonized polymer dots, photoinduced reactions, photocatalysis

## Abstract

Seeking clean energy as an alternative to traditional fossil fuels is the inevitable choice to realize the sustainable development of the society. Photocatalytic technique is considered a promising energy conversion approach to store the abundant solar energy into other wieldy energy carriers like chemical energy. Carbon dots, as a class of fascinating carbon nanomaterials, have already become the hotspots in numerous photoelectric researching fields and particularly drawn keen interests as metal‐free photocatalysts owing to strong UV–vis optical absorption, tunable energy‐level configuration, superior charge transfer ability, excellent physicochemical stability, facile fabrication, low toxicity, and high solubility. In this review, the classification, microstructures, general synthetic methods, optical and photoelectrical properties of carbon dots are systematically summarized. In addition, recent advances of carbon dots based photoinduced reactions including photodegradation, photocatalytic hydrogen generation, CO_2_ conversion, N_2_ fixation, and photochemical synthesis are highlighted in detail, deep insights into the roles of carbon dots in various systems combining with the photocatalytic mechanisms are provided. Finally, several critical issues remaining in photocatalysis field are also proposed.

## Graphical abstract

Photocatalytic technique is considered a promising energy conversion passway to alleviate energy crisis and environmental issues. Carbon dots as emerging photocatalysts or cocatalysts with outstanding physicochemical properties are widely applied in photocatalytic hydrogen generation, CO_2_ reduction, nitrogen fixation, pollutant degradation or organic synthesis, favoring a sustainable lifestyle based on photocatalytic techniques in the future.

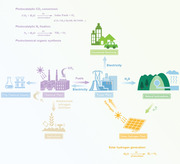

## 1 Introduction

**Scheme 1:**
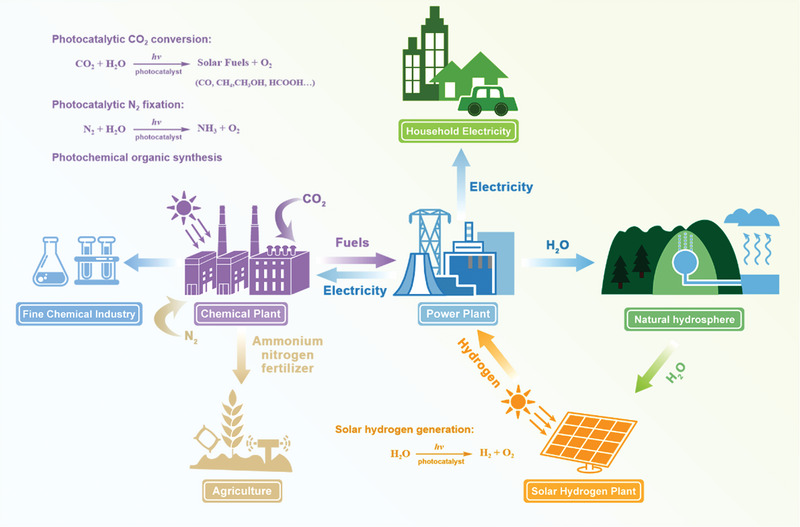
Sustainable lifestyle based on photocatalytic techniques in the future.

Rational exploration and utilization of energy resources have always been the permanent theme of human civilization. After the industrial revolution, the rapid increasing dependence on fossil fuels has brought serious global environment deterioration and ecological disequilibrium. By 2050, the global energy consumption is estimated to be double that of nowadays, yet fossil energy sources like coal or natural gas alone will not be able to meet the demand and cause further pollution.^[^ ^1^ ^]^ Therefore, seeking for the development of sustainable clean energy resources and achieving carbon neutrality to alleviate environmental issues have already become worldwide priorities. Along with the innovative discoveries and ongoing breakthroughs in the frontiers of semiconductor research, semiconductor‐based photocatalysis technologies have also made great strides, especially in solar energy‐fuels conversion and high value‐added chemicals synthesis.^[^ ^1^, ^2^ ^]^ Numerous semiconductors have been widely applied to carry out various photocatalytic reactions containing hydrogen generation, CO_2_ reduction, nitrogen fixation, pollutant degradation or organic synthesis, for addressing energy shortage and environmental crisis.^[^ ^3^ ^]^ Imagine the future where photocatalytic technology would be popularization, energy supplies, and fine chemical synthesis are becoming sustainable via hydrogen power plants and clean chemicals plants in a cyclic format, as presented in **Scheme** **1**.

Conceptually, the typical photocatalytic process includes three steps, consisting of photon absorption, charge migration, and surface chemical reactions.^[^ ^4^ ^]^ The initial light absorption involves that the semiconductors would absorb photons with energy equal to or greater than the bandgap, thus to excite electrons from the valence band (VB) of the semiconductor to the conduction band (CB), while photogenerated holes are generated in VB. Immediately afterward, the photoexcited electrons and holes migrate to the semiconductor surface and are engaged in light‐driven redox reactions with the substrate, respectively. Hence, an ideal photocatalyst is supposed to possess wide light‐harvesting range, strong optical absorption, prolonged carrier lifetime, and superior electron‐transfer efficiency.^[^ ^5^ ^]^

Carbon dots (CDs), as a sort of burgeoning photoluminescent carbon‐based nanomaterials, represent bright prospects in biomedicine,^[^ ^6^ ^]^ sensors,^[^ ^7^ ^]^ optoelectronic devices,^[^ ^8^ ^]^ etc. according to their facile fabrication, tunable optical properties, high photochemical stability, outstanding photoelectronic properties, low toxicity, and good solubility.^[^ ^9^ ^]^ Furthermore, CDs are also considered as a class of metal‐less photocatalyst with infinite potential, due to the abundance of precursors and various synthetic approaches, leading to the adjustable surface functionalization and designable energy‐level configuration.^[^ ^5^, ^10^ ^]^ To show the distinctive advantages of CDs as photocatalyst, we list a table that provides a direct comparison of the properties of CDs and other types of common photocatalysts containing organic dyes, semiconductor quantum dots, porous framework materials, and carbon nitride.^[^ ^1^, ^11^ ^]^ (**Table** **1**) Herein, we introduce the advances of CDs‐based photoinduced chemical reactions in recent years. First, we give the classification, structural and optical properties of CDs, then discuss the mechanisms and applications of CDs as photocatalyst in photoinduced chemical reactions with examples. Finally, we summarized the existing deficiencies and further propose a superficial perspective on the progress of CDs‐based photocatalysis, based on our best knowledge.

## 2 Classification and Corresponding Microstructures of CDs

**Scheme 2:**
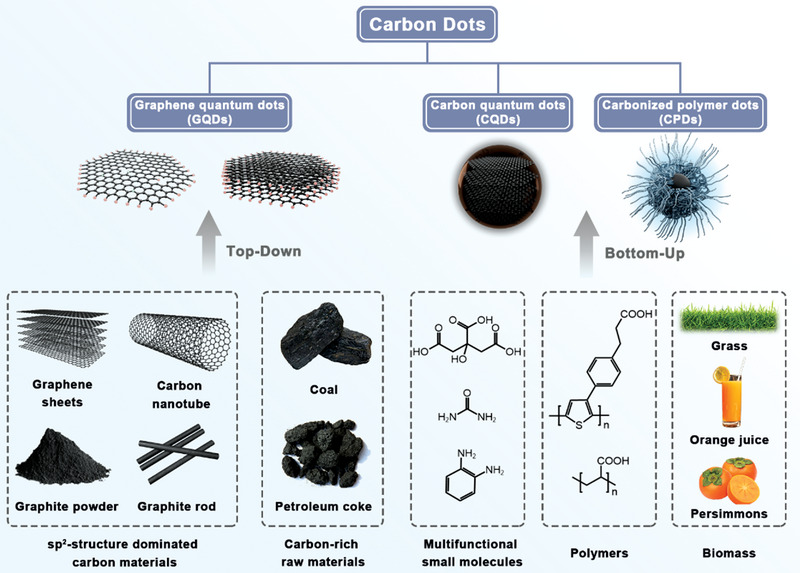
Classification of CDs: GQDs, CQDs, and CPDs, and general synthesis approaches with corresponding precursors.

CDs are an essential class of carbon‐based nanomaterials with different apparent structural properties (morphology and crystallinity) and tunable chemical properties. Due to their complex diversity, it is hard to provide an accurate definition and classification of CDs.^[^ ^12^ ^]^ Along with the in‐depth investigation and detailed characterization, we gradually have a distinct cognition to CDs. In previous report, Cayuela et al. divided CDs into graphene quantum dots (GQDs), carbon nanodots (CNDs), and carbon quantum dots (CQDs).^[^ ^13^ ^]^ We furtherly came up with the concept of carbonized polymer dots (CPDs) afterward and made a distinction from other CDs.^[^ ^9e^ ^]^ In this review, based on plenty of previous reports, we are more likely to classify CDs mainly into the following three categories: GQDs, CQDs, and CPDs (**Scheme** **2**).

### 2.1 Graphene Quantum Dots

GQDs normally refer to the nanoparticles (NPs) or fragments split from bulk carbon skeleton such as carbon nanotube, carbon fibers, graphite powder, graphene oxide.^[^ ^14^ ^]^ GQDs are anisotropic, with lateral diameter (less than 10 nm) larger than the height in vertical direction.^[^ ^7^, ^15^ ^]^ They possess typical sp^2^‐structured carbon cores, and constitute of single or several graphene layers which are connected by functional groups on edges of GQDs.^[^ ^16^ ^]^ The existence of these functional groups results in a unique edge effect of GQDs. Moreover, the quantum confinement effect (QCE) dominated by the size of GQDs and isolated *π*‐domains inside together with the edge effect synergistically impact the photoluminescent property of GQDs.^[^ ^17^ ^]^

### 2.2 Carbon Quantum Dots

CQDs are highly crystalline sp^2^–sp^3^ hybrid carbon structures in quasi spherical profiles, and the lattice fringes can be obviously observed by high resolution transmission electron microscopy (HR‐TEM).^[^ ^9e^ ^]^ Numerous functional groups are distributed on the surface of CQDs. Notably, CQDs possess representative intrinsic state luminescence, and the emission peak of CQDs shifts easily following the variation of sizes based on the QCE.^[^ ^18^ ^]^

### 2.3 Carbonized Polymer Dots

CPDs are normally interpreted as a hybrid structure consisting a highly crosslinked or slightly graphitized hydrophobic carbon core and external polymer chains with hydrophilic groups.^[^ ^9^, ^19^ ^]^ CPDs are generally derived from polymers or organic molecules rich in functional groups via the procedures of dehydration, condensation, crosslinking, and carbonization.^[^ ^20^ ^]^ In terms of different degrees of carbonization, our group furtherly classified the cores of CPDs in four submodalities, including amorphous/crystalline completely carbonization cores, paracrystalline carbon structure composed of tiny carbon clusters surrounded by polymer frames and highly dehydrated crosslinking polymer networks.^[^ ^9e^ ^]^ Compared to aforementioned CQDs or GQDs, the functional groups on precursors lead to a higher heteroatom content of CPDs such as oxygen or nitrogen, which is beneficial for applications as catalysts, sensors or photoluminescent building blocks with high photoluminescence quantum yield (PLQY).^[^ ^21^ ^]^

**Table 1:** Comparison of properties of CDs with multiple common photocatalysts

Property	Materials					
	CDs	Organic dyes	Semiconductor quantum dots	Porous frameworks	g‐C_3_N_4_	Transition metal oxides
Size	1–10 nm	<2 nm	2–10 nm	10–10^3^ nm	10^2^–10^3^ nm in 2D	20–10^3^ nm
Structure	Amorphous or crystalline	Molecular	Crystalline	Crystalline	Crystalline	Amorphous or crystalline
Solubility	High	High	High	Low	Low	Low
Toxicity	Low	Low–high, depending on molecular structure	Low–high, depending on heavy metal component	Low–high, depending on heavy metal component	Low	Low–high
Stability	Stable	Poor thermal stability and photobleaching	Stable	Poor stability to multiple external stimulus	Stable	Stable
Exc. range	UV–vis	UV–NIR	UV–vis	UV–vis	UV–vis	UV
Emission	UV–NIR	Vis–NIR	Vis–NIR	Vis–NIR	Vis	–
Abs. range	UV–NIR	UV–vis	UV–vis	UV–NIR	UV‐Vis	UV
PLQY	5–90%	5–100%	10–100%	0–90% most in solid state	<10% without modification	–
Typical synthetic method	Hydrothermal solvothermal	Organic synthesis	Hot injection and solution‐epitaxy method	Solvothermal	Pyrolysis	Solid‐state synthesis
Cost	Low	Low–high	High	High	Low	Low

## 3 Synthetic Methods

The direct fabrication of CDs is primarily divided into two directions: top‐down and bottom‐up (**Table** **2**). More specifically, the top‐down pathway dissociates the bulk carbon systems into nanoscale fragments via chemical oxidation, electrochemical etching or physical approaches.^[^ ^9d^ ^]^ Correspondingly, the bottom‐up pathway for CDs synthesis is commonly considered as the process in which molecule monomers or polymers as precursors form crosslinking polymer‐like structures through dehydration, condensation, and further local carbonization.^[^ ^9^, ^19^, ^20^ ^]^ Particularly, heteroatom doping is also a common strategy to optimize the optical and photoelectrical properties during CDs synthesis, thus to match the demands for applications.^[^ ^10b^ ^]^

**Table 2:** Synthetic methods of CDs

Classification	General raw materials	Synthetic approaches	Reaction conditions	Advantages	Disadvantages
Top‐down	Bulk carbon systems (carbon nanotube, carbon fibers, graphite powder, graphene oxide, etc.) or carbon‐rich raw materials (coal, petroleum coke)	Laser ablation	Ar atmosphere, laser	High efficiency	Wide size distribution of CDs, complex operation, high energy consumption
		Arc discharge	Ar atmosphere, arc discharge	High efficiency	Wide size distribution of CDs, complex operation, high energy consumption
		Nanolithography	Electron beam lithography, plasma treatment	Narrow size distribution, high yields of CDs	Complex operation, expensive equipments
		Mechanical milling	Mechanical ball‐milling in shaking jars	Reduced reaction times, high yields of CDs, eco‐friendliness.	Low PLQY of CDs
		Chemical oxidation	Strong oxidant	Excellent hydrophilicity and crystallinity of CDs	Strong oxidizing or acidic/alkaline waste
		Electrochemical exfoliation	Impressed voltage	High efficiency, high yields, excellent crystallinity of CDs	High energy consumption
		Metal–graphite intercalation	Potassium intercalation, ultrasonic treatment	High efficiency	Wide size distribution of CDs
		Photoinduced Fenton reaction	Strong oxidant, UV light irradiation	Large‐scale fabrication	Strong oxidizing waste
Bottom‐up	Multifunctional small organic molecules, polymers, or biomass materials.	Combustion	High temperature heating	Facile operation, solovent free	Low PLQY, low yield, wide distribution of CDs
		Pyrolysis	High temperature heating	Facile operation, solovent free, high efficiency	Low PLQY, wide distribution of CDs
		Ultrasound‐assisted	Ultrasonic treatment	Facile operation	Time‐consuming
		Microwave‐assisted	Microwave heating	Facile operation, reduced reaction times, high efficiency	Wide size distribution of CDs
		Hydrothermal/solvothermal	High pressure, high temperature heating in enclosed environment	Facile operation, wide precursor selectivity, superior PLQY	Time consuming, low yield of CDs

### 3.1 Top‐Down Methodology

In the top‐down fabrications of CDs reported so far, the adopted precursors are mainly sp^2^‐structure dominated carbon materials including graphite rod, carbon fiber, carbon nanotube, graphene oxide or some other carbon‐rich raw materials like coal, petroleum coke.^[^ ^14^, ^18^, ^22^ ^]^ Exfoliation or disintegration of the preceding large carbon systems occurs via physical methods like laser ablation,^[^ ^23^ ^]^ arc discharge,^[^ ^22a^ ^]^ nanolithography,^[^ ^14^, ^24^ ^]^ mechanical milling,^[^ ^25^ ^]^ or chemical methods consisting electrochemical exfoliation,^[^ ^18^, ^22^, ^26^ ^]^ chemical oxidation,^[^ ^14^, ^22^, ^25^, ^27^ ^]^ metal–graphite intercalation,^[^ ^28^ ^]^ and special reactions such as photoinduced Fenton reaction.^[^ ^29^ ^]^ In the process of disintegration, these bulk carbon systems are split into nanoscale fragments with carboxyl and hydroxyl groups located on its surface. CQDs and GQDs are usually the products of top‐down method, which generally possess regular structures and controllable sizes. However, top‐down method has several drawbacks: physical methods often involve specific equipment or complex nanotechnology operations, while chemical oxidation methods generate polluting exhaust gases and strong oxidizing or acidic/alkaline waste liquid during the synthesis procedure.

**Figure 1:**
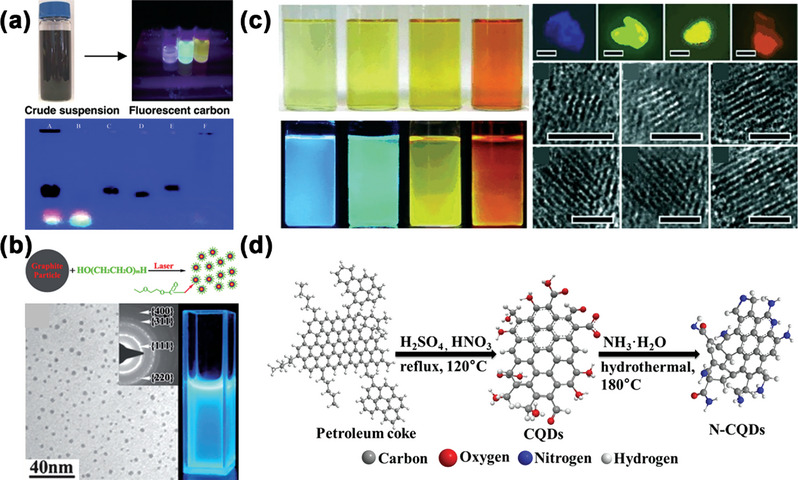
a) Optical photograph of separated carbon NPs and electrophoretic profile under 365 nm UV light. Reproduced with permission.^[^ ^22a^ ^]^ Copyright 2004, American Chemical Society. b) TEM image and photograph of the CDs prepared by laser ablation under 365 nm UV light. Reproduced with permission.^[^ ^23b^ ^]^ Copyright 2009, The Royal Society of Chemistry. c) Photographs (under room light and UV light) and HR‐TEM images of CDs solutions with different diameters fabricated by alkali‐assisted electrochemical exfoliation method. Reproduced with permission.^[^ ^18^ ^]^ Copyright 2010, Wiley‐VCH. d) Diagram for the preparation of CQDs and N‐CQDs from petroleum coke. Reproduced with permission.^[^ ^22c^ ^]^ Copyright 2014, Elsevier.

In 2004, Scrivens's group first separated fluorescent carbon NPs component derived from arc discharge soot of single‐walled carbon nanotubes, which purified by electrophoretic method (**Figure** **1**a).^[^ ^22a^ ^]^ Later on, Du and co‐workers proposed effective approaches to fabricate CDs with strong and tunable emission by laser ablation on suspension of carbon materials in organic solvents.^[^ ^23b^ ^]^ The as‐synthesized samples were furtherly treated by refluxing in strong oxidizing acid to introduce carboxyl functional groups to improve the hydrophilicity. Then, the CDs were passivated by polymers to obtain higher PLQY and stability (Figure 1b). Moreover, mechanical approaches have been widely applied in chemical synthesis because of the large‐scale yields, mild conditions and eco‐friendly synthesis procedure. Wang et al. reported a facile way to fabricate CQDs by high energy ball‐milling with activated carbon and KOH as precursors.^[^ ^25^ ^]^ By controlling the ball size, collision frequency, grinding time, and other parameters, CQDs in narrow size distributions with multiple desired optical properties were obtained.^[^ ^30^ ^]^ In 2010, Kang's group proposed an alkali‐assisted electrochemical exfoliation method for CQDs synthesis.^[^ ^18^ ^]^ Graphite rods were employed as electrode materials, the current triggered the breakage of chemical bonds and fragments exfoliated from the electrode into solution, thus to form CQDs with typical graphite lattices (Figure 1c). Another common strategy utilized for bond fracture is chemical oxidation through hydrothermal or solvothermal process under strong oxidizing agents (concentrated nitric acid, sulfuric acid or H_2_O_2_). Our group reported a one‐step solvothermal approach from graphene oxide, and the as‐synthesized GQDs possessed strong green PL with PLQY up to 11.4%.^[^ ^27^ ^]^ Hu et al. utilized H_2_O_2_ to generate active hydroxyl radicals to remove amorphous carbon in coal, thus to fabricate fluorescent CDs which presented great performance in photocatalysis.^[^ ^22d^ ^]^ Likewise, Wu et al. developed aqueous‐soluble CQDs from petroleum coke, which were primarily oxidized by H_2_SO_4_ and HNO_3_, and further functionalized by hydrothermal ammonia operation (Figure 1d).^[^ ^22c^ ^]^

### 3.2 Bottom‐Up Methodology

On the contrary side of top‐down, the bottom‐up approach for CDs synthesis is mainly based on multifunctional small molecules, polymers or biomass materials, which possess abundant functional groups consisting of amino groups, hydroxyl groups, carboxyl groups, sulfhydryl groups or vinyl groups.^[^ ^20^ ^]^ The strategies of CDs synthesis according to bottom‐up approach can be subdivided into combustion, pyrolysis, ultrasound‐assisted, solvothermal or hydrothermal, and microwave‐assisted method. Under the high pressure or elevated temperature reaction conditions, the precursors tend to dehydrate and crosslink to form a carbonized structure.^[^ ^9d,e^ ^]^ Although the top‐down synthesis was earlier developed, the bottom‐up method revealed unique merits including convenient fabrication process, low costs, wide variety of raw materials and the as‐synthesized CDs possess facile tunable fluorescent emission with higher PLQY, thus to be widely investigated afterward.

**Figure 2:**
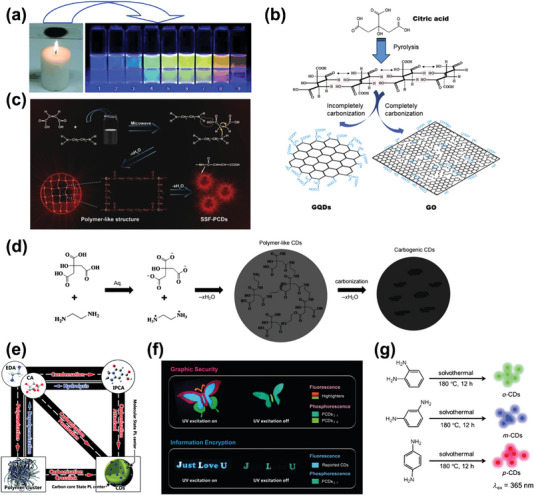
a) CDs with multicolor fluorescence obtained from the combustion soot of candles. Reproduced with permission.^[^ ^31^ ^]^ Copyright 2007, Wiley‐VCH. b) Schematic diagram of the synthesis of GQDs and graphene oxide from CA by pyrolysis. Reproduced with permission.^[^ ^32b^ ^]^ Copyright 2012, Elsevier. c) The synthetic route using maleic acid and EDA to form CPDs with possible polymer‐like structure. Reproduced with permission.^[^ ^35^ ^]^ Copyright 2017, Wiley‐VCH. d) Synthetic route of hydrothermally synthesize CDs from CA and EDA: from ionization to condensation, polymerization, and carbonization. Reproduced with permission.^[^ ^21a^ ^]^ Copyright 2013, Wiley‐VCH. e) Schematic diagram of the formations of molecular state PL center and carbon core state PL center in CDs prepared from CA and EDA. Reproduced with permission.^[^ ^36^ ^]^ Copyright 2015, The Royal Society of Chemistry. f) Phosphorescence of CPDs hydrothermally synthesized from polyacrylic acid and EDA. Reproduced with permission.^[^ ^37b^ ^]^ Copyright 2018, Wiley‐VCH. g) Syntheses of green, blue, and red emissive CDs prepared by ethanol solvothermal method. Reproduced with permission.^[^ ^40^ ^]^ Copyright 2015, Wiley‐VCH.

The preparation of CDs by combustion is operated by collecting the soot from burning candles or natural gas, refluxing in HNO_3_ and then obtained by dialysis or centrifugation (**Figure** **2**a).^[^ ^31^ ^]^ Similar to the combustion method, the pyrolysis approach means a direct condensation and further decomposition of organic precursors during the nonenclosed thermal process, which involves irreversible transformations both in physical phase and chemical constitutions.^[^ ^15^ ^]^ Most of the functional groups in precursors will decompose into H_2_O, NH_3_ or CO_2_, which make the as obtained CDs possess low heteroatoms doping content, resulted in poor yield and low PLQY. Dong et al. synthesized GQDs by pyrolyzing citric acid (CA) at 200 °C, then added into NaOH solution dropwise with vigorous stirring (Figure 2b).^[^ ^32^ ^]^ The obtained GQDs exhibited blue emission in aqueous solution under UV illumination which can be quenched by oxidative free chlorine in water, thus to be used as a facile detector. Microwave‐assisted strategy employed in CDs synthesis can be simply considered as the conversion driven by interactions between carbonous molecules and external microwave fields in confined space.^[^ ^33^ ^]^ The first exploited microwave‐assisted preparation method treated polyethylene glycol as raw material, yielded uniformly spherical CDs with diameters ≈5 nm and unapparent lattices.^[^ ^34^ ^]^ Shao et al. synthesized matrix‐free polymer‐like CPDs with red‐emitting solid state fluorescence based on ethylenediamine (EDA) and maleic acid through microwave operation (Figure 2c).^[^ ^35^ ^]^

Hydrothermal and solvothermal methods are the majority in bottom‐up CDs synthesis. The precursors are generally heated in closed autoclaves with the existence of water or other solvent under a temperature higher than the boiling point to form a closed hyperbaric environment, thus to drive the crosslinking and condensation reaction. Zhu et al. took CA and EDA as precursors and first hydrothermally synthesized CPDs with an unprecedented PLQY up to 80% (Figure 2d).^[^ ^21a^ ^]^ This CA–EDA CPDs showed strong UV absorption and an optimal emission at 443 nm. We attributed such marvelous PL of CPDs to the surface molecule state, to wit the imidazo [1,2‐a] pyridine‐7‐carboxylic acid, which was the condensation product of hydrothermal reaction, demonstrated in our following research (Figure 2e).^[^ ^36^ ^]^ Similarly, polymers or natural biomass are also common raw materials for CDs fabrication by hydrothermal method.^[^ ^37^ ^]^ For instance, our group proposed a route for synthesizing room‐temperature phosphorescence (RTP) CPDs from polymers. Based on the computational predictions, Tao et al. designed a model system derived from polyacrylic acid and EDA and the CPDs represented an optimized visible phosphorescence over 6 s (Figure 2f).^[^ ^37b^ ^]^ In addition, Ge et al. first utilized derivatives of conductive polymers polythiophene to prepare red emission CDs and developed applications in photoacoustic imaging and thermal theranostics.^[^ ^38^ ^]^ Grass, orange juice, cocoon silk, persimmons, and numerous other biomass materials rich in saccharide and natural polymers have successfully fabricated into CDs via the treatment of hydrothermal, which was applied in metal ion sensing, bioimaging, and other fields.^[^ ^39^ ^]^ The synthesis procedure of solvothermal and hydrothermal method is basically the same, except the solvent is changed from water to ethanol, formamide, dimethylformamide or glycerol. In 2015, Lin's group reported the synthesis of CDs with stable and tunable RGB fluorescence using isomers of phenylenediamines as precursors and ethanol as solvent, with following purification using silica gel column chromatography (Figure 2g).^[^ ^40^ ^]^

### 3.3 Heteroatom Doping

Heteroatoms doping is a persuasive method to regulate the optical properties and internal electron density distribution, meanwhile modify the bandgap and promote catalytic performance. Nitrogen atoms have similar atomic size to carbon atoms and have five valence electrons, so they often act as electron‐rich dopants in CDs in forms of pyridinic nitrogen, pyrrolic nitrogen or graphitic nitrogen.^[^ ^41^ ^]^ The electron pairs of nitrogen atoms occupy the *π* orbitals of carbon system, raising the integral electron density and resulting in a lower HOMO level and a wider bandgap.^[^ ^10b^ ^]^ Moreover, nitrogen doping in the core of CDs was reported to enhance the performance of photocatalysis through energy‐level engineering and elevated charge transfer efficiency.^[^ ^41a^ ^]^ On the contrary, boron is generally used as an electron‐deficient dopant in CDs, it will average out the charge of the carbon system and cause a decrease in charge density due to the presence of empty *π* orbitals of boron atoms. Interestingly, Wang et al. proposed novel magnetic CDs for safe magnetic resonance imaging. The magnetic property of CDs was generated by introducing vacancies and boron‐based monomers as substitutional defects.^[^ ^42^ ^]^ The atomic size and electronegativity of sulfur atoms differ significantly from that of carbon, making them relatively difficult to insert into the carbon lattice of CDs, mostly in the form of sulfhydryl located on the edges or outer surfaces. Xu et al. hydrothermally prepared a kind of S‐doped CDs using sodium citrate and sodium thiosulfate as raw materials, and achieved a highest PLQY of 67% among the reported S‐doped CDs.^[^ ^43^ ^]^ Also, the S‐doped CDs have demonstrated higher photocatalytic activity than undoped CDs through a photodegradation experiment operated.^[^ ^44^ ^]^

It is remarkable that examples of fluorine, selenium, silicon doping CDs have also been reported, for diverse applications based on their unique properties. Jiang and co‐workers solvothermally synthesized a fluorine and nitrogen codoped CDs from CA and urea with NH_4_F as dopant.^[^ ^45^ ^]^ On account of the highest electronegativity of fluorine, its strong electron withdrawing tendency decreased the energy level, which presented a red‐shifted excitation and emission wavelengths, thus to produce such UV–NIR responsive CDs. Li et al. synthesized Se‐CQDs with high yield by low temperature treatment of selenocysteine, which has the function of eliminating free radicals in the organism.^[^ ^46^ ^]^ According to the special core–shell microstructure of CPDs, Pan et al. developed the CPDs with hard core and soft polymer chain shell which prepared from CA and (3‐aminopropyl)‐triethoxysilane, directly employed in optical coating film with high transmittance, high hardness, and good flexibility.^[^ ^47^ ^]^

Besides the above nonmetal elements doping in CDs, introducing metal ions or constructing metal–carbon bond can also bring surprising properties optimization. Metal‐doped CDs are mainly derived from organometallic complexes or metallic salts compounding with carbon precursors under one‐step hydrothermal or pyrolysis. Wang et al. fabricated Zn‐doped CDs from zinc gluconate via a direct pyrolysis and purified by dialysis.^[^ ^48^ ^]^ The zinc content in the CDs was of great importance for biocompatibility and osteogenic capability. Another Mg‐doped CDs showed similar ability in guiding osteogenic differentiation.^[^ ^49^ ^]^ Moreover, the synergy in some specific dual metal doped CDs, as the Co, Fe codoped CDs reported by Yang et al., leading to better charge migration and resulting in excellent electronic catalytic activity.^[^ ^50^ ^]^

### 3.4 Machine‐Learning in CDs Design and Synthesis

In the traditional sense of chemical synthesis, constant trial and error by controlling synthetic variables is the mainstream which cause high proportion of unnecessary time consuming and materials waste.^[^ ^30^, ^51^ ^]^ Following the swift evolution of computational science and artificial intelligence, a robust tool for dealing huge amount of data computation, machine learning (ML), has been combined with many different researching fields to overcome the existing obstacles.

**Figure 3:**
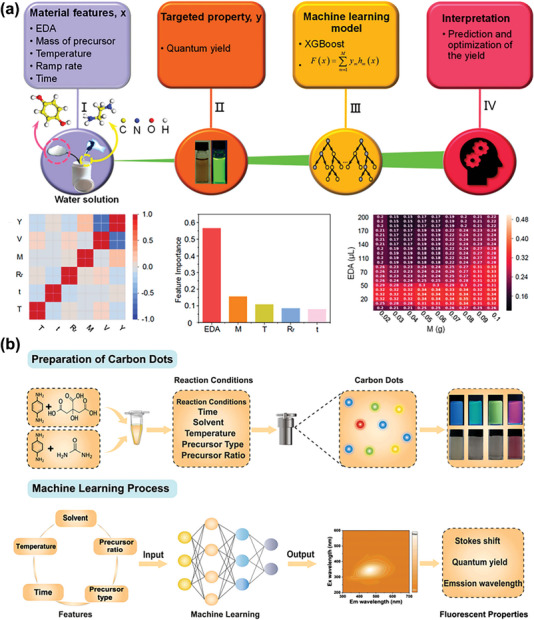
a) Application of ML for guided synthesis of CDs. Reproduced with permission.^[^ ^52^ ^]^ Copyright 2020, American Chemical Society. b) Machine learning‐assisted multicolor CDs synthesis workflow: preparation of multicolor CDs to establish training datasets for ML and predicting the PL properties of multicolor CDs with the ML model. Reproduced with permission.^[^ ^53^ ^]^ Copyright 2022, Wiley‐VCH.

Han et al. introduced ML into the high performance fluorescent CDs manufacture aimed to accelerate the exploration period and reduce the experimental cost.^[^ ^52^ ^]^ They selected five experimental parameters as the input features including volume of EDA, mass of precursor, reaction temperature, ramp rate, and reaction time and each parameter revealed a low linear correlation with the others, which confirmed the effectiveness of feature selection (**Figure** **3**a). Several regression models were evaluated by a nested cross validation method, in order to speculate QY more precisely. The trained ML model demonstrated that the superior optical properties were tightly correlated with the volume of alkaline catalyst and the mass of precursors. Based on the analysis of the model and accumulated experimental data, authors successfully synthesized green fluorescent CDs with highest QY up to 39.3%. Currently, inspired by the influencing factors proposed in previous investigation about PL behaviors of CDs, Huang and colleagues employed a data‐driven ML strategy to fabricate multicolored fluorescent CDs, as shown in Figure 3b, and then developed its application of information encryption which reflected in 36‐colcored QR code. They prepared multicolor CDs by controlling conditions artificially to establish training datasets for ML.^[^ ^53^ ^]^ Correspondingly, the ML model can feedback the predictions PL properties (Stokes shift, QY, emission wavelength) by studying the relationship between the selected synthesis parameters. The popularization of ML in CDs synthesis prediction and optimization successfully accelerates the research progress of CDs, bridging the vacuum between theory and practice.^[^ ^54^ ^]^

## 4 Optical and Photoelectrical Properties of CDs

### 4.1 Optical Absorption

**Figure 4:**
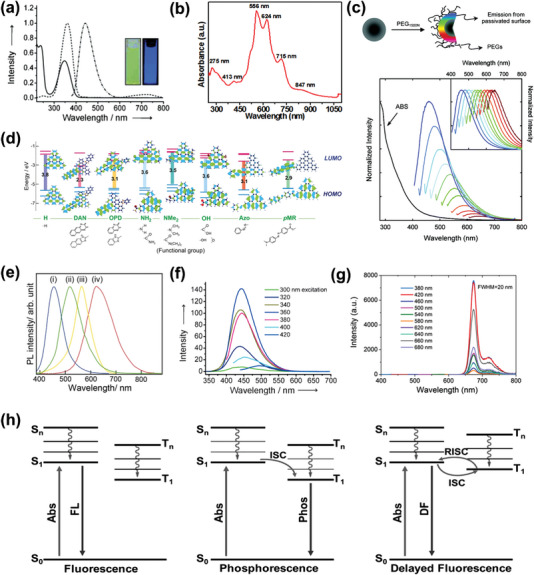
a) UV–vis absorption spectra (solid line) of CDs in aqueous solution hydrothermally synthesized from CA and EDA. Reproduced with permission.^[^ ^21a^ ^]^ Copyright 2013, Wiley‐VCH. b) UV–vis absorption spectra of F‐doped CDs prepared by solvothermal method. Reproduced with permission.^[^ ^45^ ^]^ Copyright 2020, Wiley‐VCH. c) Schematic illustration of surface passivation and PL emission spectra of PPEI‐EI passivated CDs in an aqueous solution. Reproduced with permission.^[23a]^ Copyright 2006, American Chemical Society. d) Predicted energy level diagrams for GQDs with different nitrogen functional groups. e) Normalized PL spectra of i) Azo‐GQDs, ii) NH2‐GQDs, iii) OPD‐GQDs, and iv) DAN‐GQDs from blue to red. Reproduced with permission.^[^ ^55^ ^]^ Copyright 2016, Wiley‐VCH. f) Excitation dependence of CDs in aqueous solution hydrothermally synthesized from CA and EDA. Reproduced with permission.^[^ ^21a^ ^]^ Copyright 2013, Wiley‐VCH. g) PL emission spectra of deep red emission CPDs with narrow FWHM. Reproduced with permission.^[^ ^57^ ^]^ Copyright 2020, Wiley‐VCH. h) Scheme of the emissive processes of fluorescence, phosphorescence, and delayed fluorescence. Reproduced with permission.^[^ ^59^ ^]^ Copyright 2020, The Royal Society of Chemistry.

According to the carbon dominant structure of CDs, CDs usually exhibit strong UV absorption with a band tail absorption extend to visible range. In general, the dominating absorption peak of most CDs located ≈250 nm ascribed to *π*–π* transitions of C=C bond, and another subdominating peak appeared in 300–400 nm belongs to the *n*–π* transitions of C=O/C=N bonds (**Figure** **4**a).^[^ ^21a^ ^]^ For some CDs with nearly full‐spectrum absorption, they may exhibit typical absorption peaks at specific locations in vis–NIR range. For example, the absorption spectrum of a F‐doped CDs (Figure 4b), it signified strong absorptions at 556 and 624 nm corresponding to C=N absorption of the pyridinic‐N and pyrrolic‐N, respectively.^[^ ^45^ ^]^ Other two absorption peaks in NIR region were ascribed to expanded sp^2^ domains connected by strong hydrogen bonds and pseudo‐hydrogen bonding interaction between the carbonyl of DMF solvent molecules and C—F bonds on the surface.

### 4.2 Photoluminescence

The most fascinating point of CDs materials is their stable, facilely tunable and multicolored fluorescence in the initial researching period. PL generally originates from the radiation transition resulting from the absorption of energy by photons. The emission wavelengths of GQDs and CQDs are closely associated with their sizes according to the QCE, the growth of GQDs or CQDs sizes exhibits red‐shifted PL features and vice versa.^[^ ^11^, ^17^ ^]^ Additionally, the PL property is also significantly influenced by postmodifications like surface passivation or functionalization. For instance, Sun et al. passivated the CDs synthesized by laser ablation with poly(propionylethyleneimine‐*co*‐ethyleneimine), generating enhanced bright multicolored fluorescence (Figure 4c).^[^ ^23a^ ^]^ The author attributed this fluorescence to the existence of surface energy traps that become emissive upon stabilization by polymer passivation. Moreover, Tetsuka et al. designed amino‐functionalized GQDs through nucleophilic substitution reactions between various amine moieties with oxygen‐functional groups on GQDs (Figure 4d).^[^ ^55^ ^]^ They discovered that primary or dimethyl amine modified GQDs revealed degraded HOMO orbitals and higher energy levels. O‐phenylenediamine, diaminonaphthalene, azo modifications on GQDs brought lower energy levels, thus obtaining multicolored luminescent CDs, which possess emissions from blue to red (Figure 4e).

The PL properties of CPDs behave slightly differently comparing with CQDs or GQDs. The PL spectrum of CPDs generally features wide full width at half maximum (FWHM), which is situated in blue or green emission region, and many CPDs have strong excitation dependence (Figure 4f).^[^ ^21^, ^56^ ^]^ The intricate polymer/carbon hybridization structure of CPDs makes it possible to contain multiple fluorescent centers within the NPs resulting in different emission energy levels.^[^ ^9^, ^36^ ^]^ Nevertheless, by varying the precursor and adjusting the reaction temperature, more CPDs with long wavelength luminescence and narrow FWHM are being developed. Recently, Liu et al. have synthesized deep red emission CPDs with an unprecedented FWHM down to 20 nm (Figure 4g).^[^ ^57^ ^]^ In detailed characterization, the conjugated *π* system of CPDs formed with N heterocycles and aromatic rings was identified as the single PL center, thus to generate such pure deep red emission with high QY. Besides, some CPDs also hold the feature of long persistent luminescence, including thermal activation delayed fluorescence (TADF) and RTP.^[^ ^37^, ^58^ ^]^ The phosphorescence is realized by the transition from the lowest triplet excited state T_1_ to the ground state S_0_, while the TADF is achieved by the transition from the lowest singlet excited state S_1_ to the ground state S_0_ after the process of the reverse innersystem crossing (Figure 4h).^[^ ^59^ ^]^

### 4.3 Upconversion Photoluminescence

**Figure 5:**
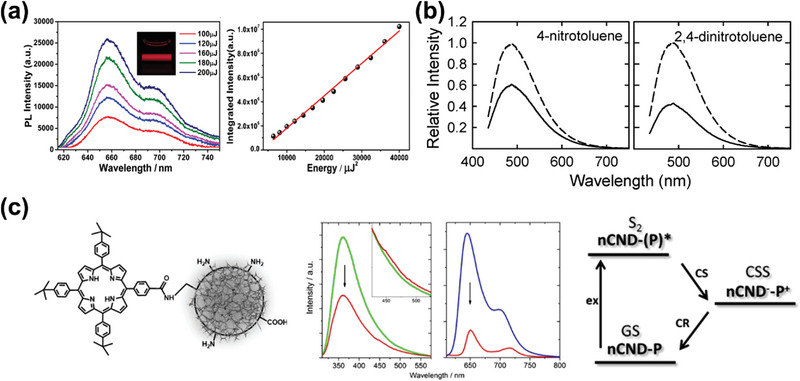
a) Upconversion PL spectra of CDs. Reproduced with permission.^[^ ^61^ ^]^ Copyright 2017, Wiley‐VCH. b) PL emission spectra of the CDs without (dashed line) or with (solid line) quenchers. Reproduced with permission.^[^ ^63^ ^]^ Copyright 2009, The Royal Society of Chemistry. c) Structure of the covalent electron donor–acceptor conjugate between CDs and tetraarylporphyrin. PL spectra of the reference porphyrin (blue), CDs (green), and the CDs‐Porphyrin (red). The energy diagram illustrating the excited‐state deactivation process. Reproduced with permission.^[^ ^64b^ ^]^ Copyright 2019, American Chemical Society.

Upconversion photoluminescence (UCPL) as a unique property of CDs has been widely utilized in photocatalysis or bioimaging fields. UCPL is a process that absorbs multiple photons at the same time and emits fluorescence with a shorter wavelength than the excitation source which conforms to an anti‐Stokes type of emission.^[^ ^11^, ^12^ ^]^ Cao et al. first observed strong two‐photon fluorescence generated by near infrared excitation in GQDs prepared by laser ablation (**Figure** **5**a).^[^ ^60^ ^]^ Subsequently, our group proposed NIR photoluminescent CPDs derived from o‐phenylenediamine and dopamine, revealed two‐photon emission under the irradiation of 800 nm femtosecond pulse laser.^[^ ^61^ ^]^ Hence, CDs with well‐behaved UCPL have great potential to be spectral converter in photocatalysis, like combining with wide band semiconductors to broaden the absorption range.

### 4.4 Photoinduced Charge Migration

Graphitized *π*‐conjugated regions distributed in CDs ensure favorable electrical conductivity. Therefore, CDs have become a rising star in photocatalysis or optoelectronics where charge transfer capability is emphasized.^[^ ^10^, ^62^ ^]^ In 2009, the fluorescence of GQDs prepared by laser ablation was first observed to be effectively quenched by electron acceptor or electron donor in solution, proposing that CDs are both excellent electron donor and electron acceptor (Figure 5b).^[^ ^63^ ^]^ Such fluorescence quenching was originated from charge migration in the excited state, demonstrated by steady‐state and time‐resolved spectroscopy afterward. For further investigating charge migration behavior as electron‐acceptor/donor in detail, Guldi et al. have employed CDs as versatile building blocks for supramolecular architectures with tailored electronic properties.^[^ ^64^ ^]^ The author synthesized covalent tetraarylporphyrin–CDs electron donor–acceptor (D–A) conjugate, and this D–A conjugate revealed a substantial emission quenching under photoexcitation in comparison to the individual components, suggesting electron migration in tetraarylporphyrin–CDs conjugate (Figure 5c). The ultrafast transient absorption spectrum of the excited conjugate reflected a similar outline to the integration of one‐electron reduced CDs and the one‐electron oxidized porphyrin, thus to confirm the charge separation, migration, and recombination process during the excited‐state deactivation of the CDs–tetraarylporphyrin conjugate.

## 5 Photocatalytic Applications of CDs

### 5.1 Photodegradation

Humanity's continuous exploration of nature has indeed made great contributions to the progress of civilization. However, during this period, an increasing amount and types of industrial and domestic pollutants have been discharged into the ecosphere, so the degradation treatment of pollutants is undoubtedly a knotty problem.^[^ ^65^ ^]^ Compared with the traditional physical adsorption method or natural degradation process, the photocatalytic degradation is more advantageous no matter in efficiency or economy. Photodegradation refers to the process in which photocatalysts generate carriers to drive redox reactions and produce reactive superoxide anion (•O_2_^−^) or •OH radicals under the excitation of specific light irradiation, realizing the conversion of environmental pollutants into harmless molecules such as H_2_O or CO_2_. In recent decades, multitudinous photocatalysts were applied to photodegradation and achieved excellent catalytic effects, especially for CDs‐based catalysts.^[^ ^66^ ^]^ In the representative works reported so far, the main targets of photodegradation can be divided into dyes, antibiotics, drug molecules, toxic metal ions, and plastic wastes, and we will give a review and discussion of typical photodegradation cases and their corresponding mechanisms in next subsections.

#### 5.1.1 Photodegradation of Dyes

**Figure 6:**
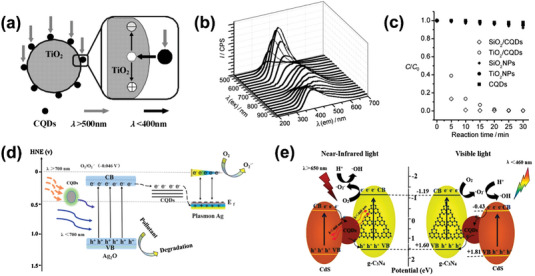
a) Possible catalytic mechanism for TiO_2_/CQDs under visible light. b) Upconverted PL properties of CQDs. c) The relationship between MB concentration and reaction time for different catalysts. Reproduced with permission.^[^ ^18^ ^]^ Copyright 2010, Wiley‐VCH. d) Schematic illustration of reaction mechanism for full spectrum degradation organic pollution on CQDs/Ag/Ag_2_O nanocomposites. Reproduced with permission.^[^ ^68^ ^]^ Copyright 2016, Elsevier. e) Schematic illustration of Z‐scheme photocatalytic mechanisms for full spectrum degradation of RhB, MB, and phenol by CSCCN nanocomposite. Reproduced with permission.^[^ ^69^ ^]^ Copyright 2020, Elsevier.

As common components in industrial wastewater, once dyes are discharged in large quantities into the aquatic environment, it may result in growth of oxygen consumption during natural degradation, leading to widespread anoxic mortality of creatures. The photocatalytic degradation for common organic dyes such as Rhodamine B (RhB), Methylene Blue (MB), Methyl Orange (MO) by various CDs‐based photocatalysts has been widely investigated so far. In 2010, Kang et al. proposed an alkali‐assisted electrochemical method to fabricate aqueous‐soluble CQDs and as‐prepared CQDs presented obvious upconversion photoluminescence (**Figure** **6**b).^[^ ^18^ ^]^ Subsequently, CQDs were hybridized with wide bandgap semiconductor TiO_2_, the composite achieved full utilization of solar spectrum. Herein, such CDs and CQDs/TiO_2_ composite were first employed for MB photodegradation (Figure 6a), thus evaluating photocatalytic activity. The composite photocatalyst reduced MB almost completely within 25 min while the individual CQDs or TiO_2_ exist scarcely any catalytic activity (Figure 6c). Similarly, upconversion property of CDs has also been widely used in later studies to construct like CPDs/Cu_2_O, CPDs/BiSbO_4_ or CQDs/Ag/Ag_3_PO_4_ complex catalysts to broaden the optical absorption, boosting the catalytic performance.^[^ ^67^ ^]^

In addition, CDs can also act as an electron transfer pathway between two photosensitive components in photocatalytic system for better separation of photoinduced electrons and holes. Chen et al. introduced CPDs into a ternary plasmonic photocatalyst CPDs/Ag/Ag_2_O to enhance photostablity and the separation of electron–hole pairs.^[^ ^68^ ^]^ According to the superior electron transfer ability and upconversion property of CPDs, this ternary photocatalyst exhibits outstanding degradation efficiency in almost full spectrum (Figure 6d). Likewise, Feng et al. synthesized a CdS/CPDs/g‐C_3_N_4_ composite (CSCCN) photocatalyst, which possesses a Z‐scheme energy‐level configuration with vis–NIR light response for the photodegradation of RhB, MB, and phenol (Figure 6e).^[^ ^69^ ^]^ The authors compared this ternary catalyst with dualistic CdS/g‐C_3_N_4_ and CPDs/g‐C_3_N_4_ by the PL spectra, electron chemical impedance spectroscopy (EIS) Nyquist plots and the transient photocurrent responses characterization. CSCCN possessed a weaker PL intensity and a smaller semicircle in EIS plots than dualistic catalysts or bare g‐C_3_N_4_ signifying a decreasing recombination of photoinduced carriers and a minor charge resistance existing in this ternary composite. The transient photocurrent responses of CSCCN showed a stronger photocurrent intensity, which obviously demonstrated prolonged lifetime of carriers.

#### 5.1.2 Photodegradation of Antibiotic Drugs

Antibiotic drugs are now widely used in the treatment of some bacterial infections, inflammation or some other diseases, however, the substance abuse problem has arisen in many countries due to the universality of antibiotics. Thousands of tons of antibiotics are released into natural environment annually, which may develop into antibiotic‐resistance pathogens as a result.^[^ ^70^ ^]^ Undisputedly, it is necessary to degrade the discharged antibiotics efficiently and photocatalysis maybe an appropriate solution to this problem.

**Figure 7:**
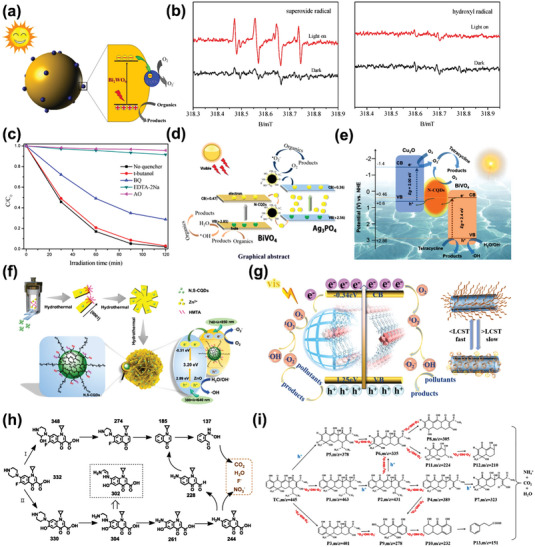
a) Schematic illustration of photocatalytic mechanism for degradation of CIP, BPA, and TC by CQDs/Bi_2_WO_6_ hybrid materials. b) ESR spectra of radical adducts trapped by DMPO in CQDs/Bi_2_WO_6_ photocatalytic system in aqueous dispersion under visible light irradiation. c) Trapping experiment of active species during the photocatalytic degradation by CQDs/Bi_2_WO_6_ composite under visible light. Reproduced with permission.^[^ ^71^ ^]^ Copyright 2015, Elsevier. d,e) Schematic illustrations of Z‐scheme photocatalytic mechanism for degradation organic pollution by Ag_3_PO_4_/N‐CDs/BiVO_4_ and Cu_2_O/N‐CDs/BiVO_4_ nanocomposites. (d) Reproduced with permission.^[^ ^74^ ^]^ Copyright 2018, Elsevier. (e) Reproduced with permission.^[^ ^75^ ^]^ Copyright 2019, Elsevier. f) Schematic illustration of photocatalytic mechanisms by N, S‐CQDs/ZnO hybrid nanoflowers under visible light. g) Schematic illustration of photocatalytic mechanisms by PCN222 encapsulating CDs as photocatalyst. h,i) Possible photodegradated pathways of CIP and TC. (f,h) Reproduced with permission.^[^ ^76^ ^]^ Copyright 2020, Elsevier. (g,i) Reproduced with permission.^[^ ^77^ ^]^ Copyright 2021, Elsevier.

In 2015, Di et al. synthesized CPDs/Bi_2_WO_6_ hybrid materials as visible‐light‐driven photocatalyst via a hydrothermal method (**Figure** **7**a).^[^ ^71^ ^]^ CPDs here as doping agents enhanced the absorption of Bi_2_WO_6_ obviously in visible light region, and the heterojunction between CPDs/Bi_2_WO_6_ is beneficial for carrier separation and migration according to PL spectra and transient photocurrent response. The signal of DMPO‐superoxide radical was observed by electron spin resonance (ESR) spectra under light irradiation (Figure 7b), and further free radical trapping experiments ensuring that •O_2_^−^ and photoinduced holes played crucial roles in photocatalytic degradation (Figure 7c). Ciprofloxacin (CIP), bisphenol A (BPA), and tetracycline (TC) were treated as target molecules of degradation which achieved effective removal within 2 h. Under a similar mechanism, the combination of ZnS/CPDs, mpg‐C_3_N_4_/GQDs, and PbBiO_2_Cl/CPDs all performed an enhanced photocatalytic degradation ability for degrading CIP or TC degradation.^[^ ^72^ ^]^

In Z‐scheme heterojunctions, photoinduced electrons in one semiconductor tend to occur recombination with holes in the other semiconductor, and this leads to accumulation of carriers at respective lower VB or higher CB in the two semiconductors.^[^ ^73^ ^]^ Thus, Z‐scheme heterojunctions generally possess higher redox potentials than traditional Type‐II heterojunctions with same composition, which is beneficial for photoinduced redox reactions. Zeng's group brought forward several CDs‐based Z‐scheme photocatalytic systems successively. Based on their reported literature, they proposed a N‐CPDs mediated Ag_3_PO_4_/BiVO_4_ photocatalyst for photocatalytic removal of TC (Figure 7d).^[^ ^74^ ^]^ Later on, they further constructed another Cu_2_O/N‐CPDs/BiVO_4_ system, the existence of N‐CPDs greatly enhanced molecular oxygen activation ability and drove force to reconstruct the local electric field, forming Z‐scheme Cu_2_O/N‐CPDs/BiVO_4_ composite catalyst (Figure 7e).^[^ ^75^ ^]^ Under the optimum condition, this composite enables to nearly completely remove TC in an hour under visible light.

In addition to the above‐mentioned examples of modifying special energy level structures by forming heterojunctions, constructing microstructures of photocatalysts is also a feasible option to enhance photodegradation efficiency. Qu et al. designed novel N, S‐CPDs/ZnO hybrid nanoflowers via a hydrothermal process.^[^ ^76^ ^]^ The N, S‐CPDs were embedded in the highly reactive facets of ZnO nanoflowers, leading to formation of 3D heterostructure (Figure 7f). Photodegradation experiments for CIP, cephalexin, and several dyes under different lighting conditions all exhibited excellent performances. Xia et al. prepared a thermoresponsive polymer functionalized porphyrin‐based Zr‐metal–organic frameworks (MOF, PCN222) encapsulating CPDs as photocatalyst.^[^ ^77^ ^]^ CPDs as a guest in PCN222 enabled to solve the problem of frequent recombination of photogenerated carriers in MOFs (Figure 7g). Besides, the grafting of hydrophilic polymer on N‐CPDs@MOFs promoted the efficiency of photogenerated charge transfer on solid–liquid interface and the enrichment of target molecules around reactive sites. Therefore, this organic–inorganic hybrid photocatalyst held a superior degradation ability for dyes or antibiotics which removed 100% MB and almost 90% TC in only 20 min. In particular, both works mentioned above further confirmed the possible degradation mechanism of CIP and TC by LC–MS, respectively (Figure 7h,i).

#### 5.1.3 Photodegradation of Toxic Metal Ions

**Figure 8:**
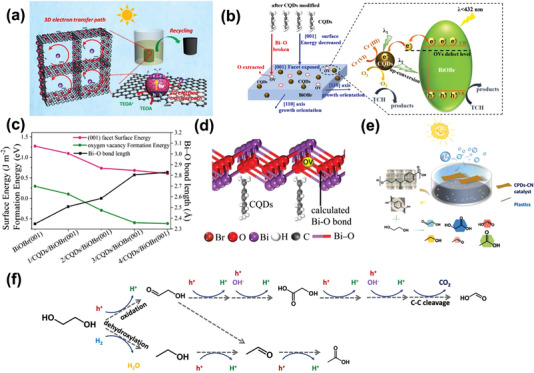
a) Schematic illustration of photocatalytic mechanism for Cr(VI) degradation by 3D graphene aerogel frameworks combined with CDs. Reproduced with permission.^[^ ^78^ ^]^ Copyright 2018, Elsevier. b) Schematic illustration of photocatalytic mechanism for Cr(VI) and TC degradation simultaneously by CQDs/BiOBr. c) Calculated Bi—O bond length, surface energy, and OV formation energy for {001} facet of CQDs/BiOBr with different CQDs qualitative content. d) Computational model of the CQDs/BiOBr {001} interface. Reproduced with permission.^[^ ^80^ ^]^ Copyright 2022, Elsevier. e) Schematic illustration of photodegradation of PET plastic hydrolysis and upcycling for chemicals production coupled with H_2_ generation. f) Proposed formation mechanism of EG derivatives during irradiation. Reproduced with permission.^[^ ^83^ ^]^ Copyright 2022, Elsevier.

The hexavalent chromium ions (Cr(VI)), which have already been listed as a primary carcinogen by WHO, exist extensively in discharged industrial wastewater and possess strong biological toxicity. Photocatalysis can effectively reduce hexavalent chromium ions to trivalent, which provides a promising treatment of detoxification for wastewater. In 2018, Xu's group formed a metal‐free 3D catalyst that combined CPDs with 3D graphene aerogel frameworks.^[^ ^78^ ^]^ This bulk composite aerogel catalyst exhibits efficient spatial separation and transportation of photogenerated charge carriers due to its interconnected networks inside, which removed over 90% of Cr(VI) within 40 min under UV–vis light irradiation, much higher than single CPDs catalysts (**Figure** **8**a). Likewise, Zhang et al. constructed CPDs‐deposited CdS nanosheets for photoreduction of Cr(VI) to Cr(III) in saline water which represented favorable stability.^[^ ^79^ ^]^ In addition to the degradation of single target mentioned, researchers began to focus on the simultaneous photodegradation of the two pollutants by virtue of the dual functionality of the catalyst. Recently, Dong and co‐workers introduced CPDs into BiOBr to modify (001) crystal facets exposed and formed surface oxygen vacancies on these facets through a hydrothermal method.^[^ ^80^ ^]^ They aimed to wipe off TC and Cr(VI) in acidic wastewater simultaneously (Figure 8b). Density functional theory was utilized to investigate the mechanisms of the growth of the dominant (001) facet and the formation of oxygen vacancies (Figure 8c). This (001) facet exposed more O atoms than other crystal facets, which exist stronger H‐bond interactions with CPDs, leading to an increased deposition of CPDs. The strong interactions between CPDs and the (001) facet of BiOBr may stretch the Bi—O bond and cause broken. In this way, O atoms were extracted from the [Bi_2_O_2_]^2+^ layers, resulting the formation of oxygen vacancies on (001) facet (Figure 8d). The best performance of this catalyst can remove 96% TC and almost 100% Cr(VI) within an hour in an acidic environment.

#### 5.1.4 Photodegradation of Plastic Waste

Besides pollutants in aqueous environment, the degradation or conversion of solid plastic waste is also a crucial problem in eco‐sustainable field. In recent years, the world's annual production of plastic has been maintained at hundreds of millions of tons.^[^ ^81^ ^]^ However, most plastics are difficult to degrade in natural conditions, and the artificial incineration or landfill treatments may produce abundant flue gas or plastic particles into the environment, thus give rise to some damage to creatures.^[^ ^82^ ^]^

Recently, Han et al. designed a metal‐free CPDs‐based photocatalyst, which realized photocatalytic poly(ethylene terephthalate) (PET) and poly(lactic acid) (PLA) recycling coupled with H_2_ generation simultaneously (Figure 8e).^[^ ^83^ ^]^ This composite catalyst is synthesized by adding as prepared graphitic carbon nitride (GCN) powders into CPDs solutions then through a hydrothermal procedure. PET or PLA plastics need to go through a pretreatment with alkaline before hydrolysis and H_2_ production. The PET plastics would be hydrolyzed into terephthalic acid and ethylene glycol (EG) first, and EG will be further upcycled into high value‐added chemicals such as ethanol, glycolic acid, acetic acid, etc. under the irradiation (Figure 8f). In the aforementioned procedure, photogenerated holes were continuously consumed, while the remaining electrons drove the reduction from H_2_O to H_2_. Through the reduced fluorescence lifetime and PL intensity, it has been revealed that the introduction of CPDs into GCN can effectively improve the carrier lifetime and facilitate carrier separation. EIS and photocurrent–time plots reflected that a proper doping ratio of CPDs in GCN enable a lower resistance and superior charge transfer ability. This work provides a novel way for plastic recycling and a strategy to convert optical energy into H_2_ fuels by photocatalysis.

### 5.2 Photocatalytic Hydrogen Generation

Due to the long‐standing reliance of human society on fossil energy, severe pollution to the environment has been caused and the greenhouse effect has been continuously aggravated. Hydrogen energy, as a perfect clean energy source, is considered as a prospective fossil‐fuel alternative due to its high energy density and absence of detrimental combustion emissions.^[^ ^4^, ^84^ ^]^ Conventional industrial approaches like steam reforming with fossil fuels or electrolysis of water for hydrogen generation involve substantial energy losses, resulting in poor efficiency. Since the Honda–Fujishima effect was discovered in 1972, scientists have dedicated their efforts to making the harsh conditions of hydrogen generation mild and controllable by photocatalysis.^[^ ^4^, ^85^ ^]^ CDs as an emerging photocatalyst revealed great potential in hydrogen evolution reaction (HER), especially when hybridizing with various semiconductors.

#### 5.2.1 Photocatalytic HER from Water

**Figure 9:**
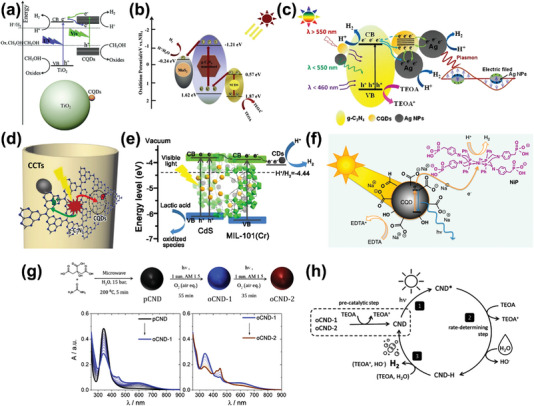
a) Schematic illustration of photocatalytic HER mechanism by CQDs‐modified P25 TiO_2_ composite. Reproduced with permission.^[^ ^86^ ^]^ Copyright 2014, The Royal Society of Chemistry. b) Schematic illustration of Z‐scheme photocatalytic HER mechanism by g‐C_3_N_4_/N‐CDs/MoS_2_. Reproduced with permission.^[^ ^88^ ^]^ Copyright 2019, Elsevier. c) Schematic illustration of photocatalytic HER mechanism by ternary plasmonic catalyst Ag/CDs/g‐C_3_N_4_. Reproduced with permission.^[^ ^91^ ^]^ Copyright 2017, Elsevier. d) Schematic illustration of photocatalytic HER mechanism by tubular CQDs‐anchored g‐C_3_N_4_. Reproduced with permission.^[^ ^94^ ^]^ Copyright 2018, Wiley‐VCH. e) Schematic illustration of photocatalytic HER mechanism by CDs/CdS modified MIL‐101. Reproduced with permission.^[^ ^95^ ^]^ Copyright 2019, Elsevier. f) Schematic illustration of photocatalytic HER mechanism by CDs hybridized with NiP molecular catalysts. Reproduced with permission.^[^ ^96^ ^]^ Copyright 2015, American Chemical Society. g) Preparation of photo‐oxidized CDs and evolution of the steady‐state absorption spectra during the photo‐oxidation transformation of CDs. h) Schematic illustration of photocatalytic HER mechanism by photo‐oxidized CDs. Reproduced with permission.^[^ ^99^ ^]^ Copyright 2021, American Chemical Society.

In pursuit of sufficient solar energy utilization, higher charge transfer efficiency, and suppressed photoexcited carrier recombination, CDs compound with semiconductors have become the dominant approach to construct heterostructures, thus to regulate the energy‐level configuration for extraordinary photocatalytic hydrogen generation performance. Zhang's group prepared CQDs modified P25 TiO_2_ composite, achieving nearly four times higher H_2_ generation rate than pure P25 under UV–vis light irradiation (**Figure** **9**a).^[^ ^86^ ^]^ Such wide band semiconductors were generally excited only by UV light illumination. The introduction of CQDs as photosensitizer broadened the absorption range of P25, therefore promoted the responsivity of composite under visible light. In addition, the CQDs enabled to extract electrons from the CB of P25, hence impeded the rapid recombination of photoinduced electrons and holes. Ternary CDs‐based catalyst like MoS_2_/CPDs/ZnIn_2_S_4_ was investigated to establish a harmonious catalytic system with synergistic catalytic effect.^[^ ^87^ ^]^ Herein, CPDs acted as an electron mediator to accelerate the fluxion of photoinduced electrons from ZnIn_2_S_4_ to MoS_2_. Moreover, another ternary Z‐scheme catalytic system, integrated by g‐C_3_N_4_ nanosheets decorated with MoS_2_ QDs and N‐CPDs, was synthesized for application in photocatalytic H_2_ generation.^[^ ^88^ ^]^ The Z‐scheme charge transfer mode of g‐C_3_N_4_/N‐CPDs/MoS_2_ signified that the excited electron in CB of N‐CPDs migrated to the VB of g‐C_3_N_4_ and counteract with the holes, then the electrons in g‐C_3_N_4_ flowed to the CB of MoS_2_ for a better catalytic performance of H_2_ (Figure 9b). The Z‐scheme photocatalytic system ensured that the photogenerated carriers were endowed with superior redox capacity in each primary bands.

Introduction of noble metal NPs has also become a common strategy to broaden the absorption in visible range of CDs‐based photocatalytic systems via surface plasmon resonance (SPR) effects. Electrons in the metallic NPs tend to collective oscillation when the NPs are excited by incident light, then relaxed into single electron–hole pairs through Landau damping, thus to produce energetic hot electrons and participate photoredox procedures.^[^ ^89^ ^]^ Zeng et al. prepared a CuNPs/CPDs composite based on a simple in situ photoreduction method.^[^ ^90^ ^]^ The optimal rate of H_2_ evolution reached 64 mmol g^−1^ h^−1^ and maintained stable in 6 cycles at least. In their following research, Ag NPs were embedded in a CPDs/g‐C_3_N_4_ composite to further widen the visible and even NIR region absorption supported by the SPR effect of Ag NPs and upconversion photoluminescence of CPDs.^[^ ^91^ ^]^ Herein, CPDs as electron acceptors and mediators, enabled to capture the redundant carriers from Ag NPs and g‐C_3_N_4_ to potentiate charge separation and avoid phototriggered corrosion from holes (Figure 9c). In this way, the mutual assistance and collaboration between the components of this catalyst brought a high HER performance of 626.93 µmol g^−1^ h^−1^ under visible light and catalytic stability.

Besides the adjustment of energy‐level configuration, synergistic effect of the CDs‐based photocatalyst morphology is also the key for affecting HER capacity. To date, CDs have been compounded in multiple semiconductor catalysts with different topological structure like nanorods, nanotubes or porous materials for photocatalytic hydrogen production.^[^ ^92^ ^]^ Kang and co‐workers systematically evaluated a series of CQDs/CdS composites with different morphologies in terms of ability to photocatalytic hydrogen production in water and seawater.^[^ ^93^ ^]^ The enhanced catalytic efficiency and stability especially in seawater were ascribed to the role of CQDs as cocatalyst, which promoted charge separation and stabilized electrons avoiding distractions from seawater. CQDs/CdS nanosheets and nanorods possessed stronger photoresponse and smaller resistance than nanospheres and other irregular particles. Zhao and colleagues fabricated a tubular CPDs‐anchored g‐C_3_N_4_ through a thermal polymerization process.^[^ ^94^ ^]^ Numerous microregion heterojunctions were constructed during the CPDs‐assisted assembling of g‐C_3_N_4_ nanotubes, which brought significant work function difference between CPDs and the CB of g‐C_3_N_4_, thereby accelerating the spatial separation and transferring of photoexcited carriers (Figure 9d). This tubular nanocomposite catalyst displayed stronger photocurrent intensity and reduced resistance compared with bare g‐C_3_N_4_ and CPDs/g‐C_3_N_4_, which is originated from the enhancement of absorption in visible light and the interaction coupling of g‐C_3_N_4_ and CPDs. Ultimately, this strategy led to a rather high hydrogen generation rate to 3538.3 µmol g^−1^ h^−1^ and a quantum yield of 10.94%. In the subsequent research, Zhang et al. successfully introduced CdS QDs and CPDs into the pores of metal organic frameworks (MIL‐101) simultaneously via a thermal process.^[^ ^95^ ^]^ The cage structures of MOFs supplied plenty of attachment sites for guest molecules, and the host–guest interactions between MOFs and CPDs/CdS would create an extremely efficient charge transportation channel (Figure 9e). CdS, as the component of photoharvest agent in this ternary photocatalyst, was excited and generated electrons in CB in response to visible light illumination. Owing to the interleaved energy‐level configuration, the photogenerated electrons gravitated toward CB of MIL‐101 and further were extracted by CPDs to expedite the mobility of electrons, then donated electrons to H^+^ to accomplish hydrogen evolution. CPDs/CdS@MIL‐101 exhibited an optimized rate for H_2_ generation several times higher than bare CdS or CdS@MIL‐101.

CDs have also hybridized with molecular catalysts and employed as photosensitizer for photocatalytic H_2_ generation. Reisner et al. first combined CPDs derived from pyrolysis of CA with a Ni‐bis‐(diphosphine) catalyst (Figure 9f), reaching an efficiency for photocatalytic H_2_ evolution of 398 µmol (g_CPD_)^−1^ h^−1^.^[^ ^96^ ^]^ Upon the illumination of UV–vis light, the photoexcited electrons transferred from CPDs to NiP molecular as catalytic center to reduce H^+^ to H_2_. However, this homogeneous catalytic system cannot be stable persistently due to the degradation of NiP molecular by oxidation products radicals of sacrificial agent. Hence, they followed up with a clean two‐electron donor system consisting of tris(carboxyethyl)phosphine and ascorbic acid, which avoided the radical‐mediated decomposition of the catalyst.^[^ ^97^ ^]^ This donor system prolonged the lifetime of CPDs/NiP composite to five days with a higher turnover number. Moreover, their following work and the work of Ladomenou's group both confirmed that N‐CPDs/cobalt‐based molecular catalysts also offered great potential in H_2_ generation.^[^ ^98^ ^]^

Apart from the preceding CDs‐based composite photocatalysts, it was worth mentioning that Jana et al. reported an unprecedented achievement for all‐in‐one photocatalytic hydrogen generation by pure CPDs gathering the dual functions as photosensitizer and photocatalyst.^[^ ^99^ ^]^ The optimized CPDs for photocatalysis were obtained from urea and CA undergoing a microwave‐assisted hydrothermal process, followed by postsynthetic modifications of photo‐oxidation (Figure 9g,h). Citrazinic acid (CZA) molecular was signified as the fluorophore fragments interacted with as‐synthesized CPDs, which were the center for photo‐oxidation. The photo‐oxidized CZA on CPDs resulted in a strong absorption enhancement in range from 400 to ≈800 nm. Furthermore, as for the mechanism, the CZA molecular boosted the rate of bimolecular electron transfer upon photo‐oxidation, which was strongly associated with the final HER activity. Such photocatalytic‐active CPDs exhibited HER activities record of 15.15 and 19.70 mmol g^−1^ h^−1^ for water and seawater, much higher than the similar catalysts for HER reported previously.

#### 5.2.2 Photocatalytic Overall Water Splitting

**Figure 10:**
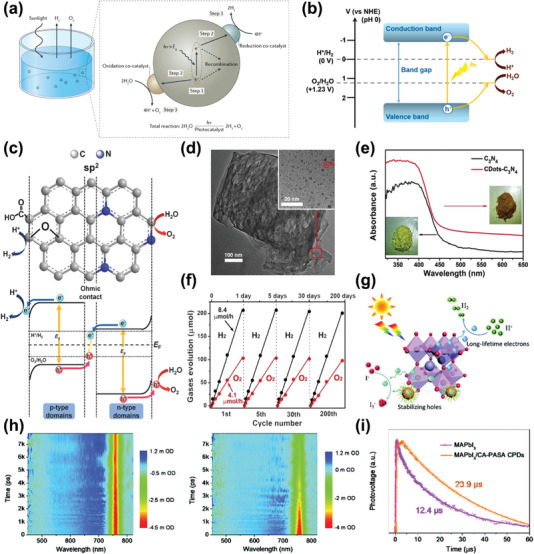
a) Schematic illustration of main processes within photocatalytic OWS. Reproduced with permission.^[^ ^85b^ ^]^ Copyright 2017, Nature Publishing Group. b) Energy diagram for photocatalytic OWS based on one‐step excitation. Reproduced with permission.^[^ ^4^ ^]^ Copyright 2020, American Chemical Society. c) Schematic illustration of the ohmic contact in N‐GQDs accelerated photocatalytic OWS. Reproduced with permission.^[^ ^101^ ^]^ Copyright 2014, Wiley‐VCH. d) TEM image of a grain of the CDs–C_3_N_4_ composite. e) UV–vis absorption spectra of C_3_N_4_ (black curve) and CDs–C_3_N_4_ (red curve) catalysts. f) Typical time course of H_2_ and O_2_ generation from water under visible light irradiation. Reproduced with permission.^[^ ^102^ ^]^ Copyright 2015, American Association for the Advancement of Science. g) Schematic illustration of photocatalytic mechanism of HI splitting by MAPbI_3_/CPDs. h) MAPbI_3_ (left) and MAPbI_3_/CPDs (right) spectrotemporal transient absorption maps excited at 400 nm. i) Transient photovoltage curves of MAPbI_3_ and MAPbI_3_/CPDs under 355 nm excitation. Reproduced with permission.^[^ ^107^ ^]^ Copyright 2020, The Royal Society of Chemistry.

Overall water splitting (OWS) into H_2_ and O_2_ directly is always a challenging task for the high Gibbs free energy up to 237.13 kJ mol^−1^, which used to be regarded as “Holy Grail” in chemistry.^[^ ^85b^ ^]^ In the past decades, prominent progress has been achieved in photocatalytic OWS along with the development of semiconductors, especially for the design of photocatalysts. As far as the thermodynamic constraints of this reaction concerned, the minimum value of the CB for the photocatalyst should be more negative than the H^+^/H_2_ energy level (0 V vs NHE, at pH = 0) to reduce protons to H_2_. On the other hand, the maximum value of the VB ought to be more positive than the O_2_/H_2_O level (+1.23 V vs NHE, at pH = 0) to drive water photo‐oxidation (**Figure** **10**a,b).^[^ ^85^, ^100^ ^]^ In other words, the photon energy required to proceed overall water splitting is at least 1.23 eV upon one‐step photoexcitation.

In 2014, nitrogen‐doped graphene quantum dots (N‐GQDs) were first reported as a visible‐light photocatalyst for OWS, and H_2_ and O_2_ was generated in 2:1 stoichiometric ratio under irradiation.^[^ ^101^ ^]^ P‐type domains and N‐type domains both existed in this N‐GQDs, connected by sp^2^ carbon clusters as ohmic contact, which were responsible for charge separation, generating H_2_ and O_2_ separately (Figure 10c). Later on, Kang's group fabricated a metal‐free CQDs/g‐C_3_N_4_ nanocomposite (Figure 10d) from low‐cost, earth‐abundant materials for solar‐driven OWS, exhibiting a marvelous solar to hydrogen (STH) energy conversion efficiency ≈2%.^[^ ^102^ ^]^ The introduction of CQDs effectively promoted absorption of this composite in visible region (Figure 10e). The optimized composite of CQDs/g‐C_3_N_4_ has represented admirable long‐term stability over 200 catalytic cycles in 200 days (Figure 10f). According to the detailed characterizations, the author inclined to a stepwise two‐electron/two‐electron process mechanism rather than the thermodynamically favorable four‐electron transfer process of O_2_ generation. The water was first oxidized into H_2_O_2_ and H_2_, then followed by a decomposition of H_2_O_2_ to O_2_ and H_2_O. Similar to photocatalytic HER, CDs‐based bandgap engineering by hybridizing with metallic semiconductors including TiO_2_ nanotube arrays, CdS, octahedral CoO, etc. has also been extensively investigated for efficient photocatalytic OWS.^[^ ^103^ ^]^ A ternary Z‐scheme photocatalyst BiVO_4_/CQDs/CdS was reported by Kang's group for visible light OWS, and CQDs here acted as solid electron mediators to supply fluent carrier‐migration pathways between adjacent semiconductors.^[^ ^104^ ^]^ Additional, Zhang et al. subsequently reported an example utilizing the SPR effect of plasmonic CuCo bimetal particles modified with CPDs to conduct OWS.^[^ ^105^ ^]^

#### 5.2.3 Photocatalytic HER from Other Precursor

Water is not the exclusive reactive precursor of photocatalytic HER, and H_2_S and HI is also desirable reactants. Recently, Zhou et al. synthesized S, N‐codoped CPDs/g‐C_3_N_4_ nanosheets via hydrothermal method to decompose H_2_S for H_2_ generation.^[^ ^106^ ^]^ Heteroatom doping in CPDs formed multiple energy‐level configurations responsible for absorption of light at different wavelengths, therefore to facilitate reaction proceeding as photosensitizer. Metal halide perovskites as emerging semiconductors are popular in the field of catalysis, and our group first reported the combination of MAPbI_3_ with CPDs for the photocatalytic HI splitting of HER (Figure 10g).^[^ ^107^ ^]^ Herein, this CPDs were hydrothermally synthesized from critic acid and p‐aminosalicylic in acidic environment, and possessed the energy level of the as‐prepared CPDs is well‐matched with MAPbI_3_. The femtosecond transient absorption spectroscopy kinetics at 760 nm became faster after hybridizing with CPDs (Figure 10h). Besides, the transient photovoltage measurement revealed a longer half‐lifetime for the composite catalyst (Figure 10i), indicating the outstanding charge separation and transfer between CPDs and MAPbI_3_. The CPDs efficiently extracted photogenerated holes from MAPbI_3_ to retard carrier recombination, which was beneficial for surface reaction, leading to high photocatalytic performance. In the case of loading Pt as cocatalyst, the highest H_2_ evolution rate equaled up to 11497 µmol g^−1^ h^−1^ and created a record STH of 2.15%.

### 5.3 Photocatalytic CO_2_ Conversion

**Figure 11:**
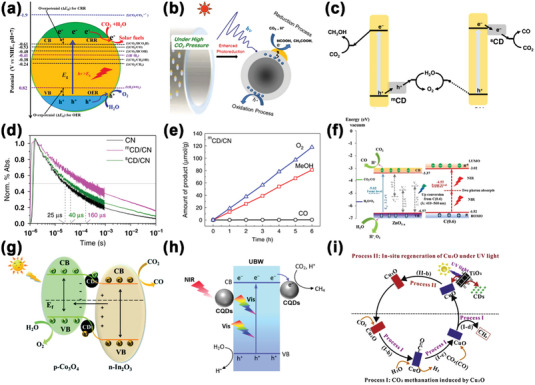
a) Schematic illustration of photocatalytic CO_2_ conversion on a semiconductor. Reproduced with permission.^[^ ^110^ ^]^ Copyright 2019, American Chemical Society. b) Schematic illustration of photocatalytic CO_2_ conversion to carboxylic acids by Au coating CDs. Reproduced with permission.^[^ ^113^ ^]^ Copyright 2014, American Chemical Society. c) Schematic illustration of photocatalytic CO_2_ reduction to methanol by the ^m^CD/CN. d) µs‐TAS decay kinetics of CN, ^m^CD/CN in water. e) The photocatalytic activity of ^m^CD/CN measured under visible light. Reproduced with permission.^[^ ^115^ ^]^ Copyright 2020, Nature Publishing Group. f) Schematic illustration of photocatalytic CO_2_ conversion to CO by ZnO_1−_ *_x_*/CDs hollow spheres. Reproduced with permission.^[^ ^118^ ^]^ Copyright 2018, Elsevier. g) Schematic illustration of photocatalytic CO_2_ conversion to CO by CDs‐MOFs composite. Reproduced with permission.^[^ ^120^ ^]^ Copyright 2022, The Royal Society of Chemistry. h) Schematic illustration of photocatalytic CO_2_ methanation by CQDs/ultrathin Bi_2_WO_6_ nanosheets. Reproduced with permission.^[^ ^121^ ^]^ Copyright 2017, Tsinghua University Press and Springer GmbH, Germany. i) Schematic illustration of photothermal coupled configuration for CO_2_ methanation by Cu/TiO_2_/CDs. Reproduced with permission.^[^ ^122^ ^]^ Copyright 2019, Elsevier.

Rapid industrial advancement and world population proliferation resulted in a tremendous increase of the continuous demand for energy. Fossil energy, as the main source of direct energy at current stage, has led to a massive emission of greenhouse gases into the atmosphere, where CO_2_ is the iconic portion. The extremely ascending atmospheric CO_2_ level has triggered ripple effects including greenhouse effect, global climate warming, and sea level rise in recent decades, posing a great threat to the global ecological system.^[^ ^108^ ^]^ As a consequence, it is necessary to explore feasible, efficient, and eco‐friendly technologies to achieve CO_2_ capture. Photocatalytic reduction from CO_2_ into CO or other valuable low‐carbon fuels seems to be a compelling and promising option.^[^ ^109^ ^]^ Theoretically, the multielectron and multiproton reduction process of CO_2_ is generally more favorable due to its relatively low thermodynamic barriers, comparing with the water reduction reaction (**Figure** **11**a).^[^ ^110^ ^]^ Nevertheless, since similar thermodynamic potentials of the reduction products CO_2_ and H_2_O, the critical challenge now is to obtain highly selective target products.^[^ ^111^ ^]^ So far, CDs‐based photocatalysts have been reported to convert CO_2_ into CO, methanol, methane, and carboxylic acids.

Pioneering report of photocatalytic CO_2_ conversion by CDs‐based catalysts mainly focused on reactions that carboxylic acid as products. In 2011, Sun and colleagues first proposed PEG‐functionalized CQDs with metal coating on surface for photocatalytic CO_2_ conversion.^[^ ^112^ ^]^ The Au or Pt coating on the surface of CQDs as cocatalyst enhanced charge separation by extracting photoexcited electrons from the surface defect states of CQDs and endowed the composite with a superior optical absorption via SPR effect. Formic acid was identified as the main product of CO_2_ photoreduction with the quantum yield of 0.3%, which was much higher than P25 as photocatalyst under the same UV irradiation. As the increase of CO_2_ pressure, concentration of CO_2_ in the aqueous solution increased, and formic acid generation increased proportionally. In their subsequent research, the gold‐doped CQDs as photocatalyst were also relatively more efficient to produce acetic acid comparing with the previous published (Figure 11b).^[^ ^113^ ^]^

In addition, CDs enable to be employed as cocatalyst compounding with various semiconductors according to its outstanding conductivity, charge migration capacity, and higher work function. MacFarlane's group synthesized a CPDs/Cu_2_O heterostructure as photocatalyst applied in CO_2_ selective conversion to methanol via ultrasonic‐assisted method.^[^ ^114^ ^]^ The introduction of CPDs slightly narrows the direct bandgap of composite to 1.96 eV, which signified a wider absorption range comparing with bare Cu_2_O (2.2 eV). The highest activity of CPDs/Cu_2_O heterojunctions for methanol conversion was up to 56 µmol g_cat_^−1^ h^−1^. Moreover, the nanocomposite also showed a strong stability in cyclic catalytic experiment. Carbon nitride (CN) as a burgeoning group of facile tunable organic semiconductors, has exhibited great potential in HER and CO_2_ reduction based on its negatively positioned CB. However, the thermodynamically similar VB position compared to the water oxidation potential and the self‐difficulties in kinetic limit the performance of CN in the water oxidation process. Hence, Wang et al. proposed an infrequent hole‐accepting ^m^CPDs to decorate CN with the target of optimizing water oxidation, leading to efficient selective CO_2_ reduction to methanol (Figure 11c).^[^ ^115^ ^]^ The ^m^CPDs/CN composite was prepared by microwave approach and the as‐synthesized ^m^CPDs were in graphite phase. Transient absorption spectroscopy (TAS) was applied to investigate the carrier dynamics of ^m^CPDs/CN and CN, and the results demonstrated an extended half‐life time from 25 µs (CN) to 160 µs (^m^CPDs/CN) (Figure 11d), indicating a suppressed charge recombination. The photocurrent response of ^m^CPDs/CN was nearly twofold higher than single CN, which manifested a promoted charge separation and the hole‐accepting property of ^m^CPDs. The average production rate of CO_2_ conversion to methanol catalyzed by ^m^CPDs/CN was 13.9 µmol g^−1^ h^−1^ under visible light irradiation in 6 h (Figure 11e), with an internal quantum efficiency (IQY) of 2.1% and marvelous selectivity of 99.6% ± 0.2%. In their recent published work, they modified the previous CN into a carbon nitride‐like polymer (FAT) through the linker/terminal group engineering.^[^ ^116^ ^]^ Oxygen atoms here went to replace some of the nitrogen linkers or terminal groups, in order to optimize the adjustable bandgap, band position, and hydrophilicity for better charge separation, adequate optical absorption, and favorable contact with aqueous solvents. Approximately, the aforesaid efficient hole‐accepting CPDs integrated with FAT polymer enabled to achieve selective CO_2_ photoreduction to methanol by water under continuous 420 nm irradiation with the IQY of 6%. CPDs as cocatalyst could extract photoexcited holes from FAT within sub‐microsecond timescale before trapped, thus to obtain improved carrier separation and elevated photoelectron density which was beneficial for CO_2_ to methanol, involving a six‐electron transfer process.

The two‐electron/two‐proton pathway of CO_2_ reduced to CO has been widely investigated in virtue of CDs‐based photocatalysts. Ong et al. operated the CO_2_ photoreduction by CPDs/protonated g‐C_3_N_4_ heterojunction photocatalyst.^[^ ^117^ ^]^ CPDs and g‐C_3_N_4_ with opposite charge properties were assembled by mutual electrostatic attraction to synthesize this heterostructured nanohybrids as photocatalyst. The optimized ratio of catalyst showed the highest activity of CO and CH_4_ evolution was found to be 58.82 and 29.23 µmol g_cat_^−1^ in 10 h. Unfortunately, the major drawback of this work is the poorly selectivity. Biswas's team designed ZnO_1‐_ *_x_*/CPDs hollow spheres served as photocatalyst over the full spectrum (Figure 11f).^[^ ^118^ ^]^ The unique hollow structure allowed the light to conduct multiple internal reflections resulting in strong absorption. Upconverted photoluminescence emissions of CPDs could efficiently excite ZnO_1‐_ *_x_*, thus to generate such wide absorption to NIR. The oxygen vacancies of ZnO_1‐_ *_x_* and anchoring CPDs jointly heightened charge transfer and CO_2_ adsorption. The best photocatalytic behavior of average CO generation rate was dozens of times higher than pristine ZnO or commercial P25. Particularly, the composite of CDs immobilized in porous materials has been proven as ideal photocatalysts featuring high specific surface area and tunable porous structure, facilitating CO_2_ adsorption and providing abundant active sites. Zhong et al. first reported a covalent organic frameworks (COFs) hosting metalloporphyrin‐based CPDs that are inside, as a heterogeneous catalyst for CO_2_ selective reduction to CO.^[^ ^119^ ^]^ COFs supplied appropriate interspace for CO_2_ adsorption, and metalloporphyrin inside were treated as CO_2_ activation sites. Likewise, Kang's group proposed the MOFs synthesized from Co_3_O_4_/In_2_O_3_ nanotubes, then modified by CQDs (Figure 11g).^[^ ^120^ ^]^ This CQDs‐MOFs composite presented a solar‐driven CO generation rate of 2.05 µmol g^−1^ h^−1^, which three times higher than Ru‐based coordination compound as catalyst, in the same condition without sacrificial agent. Interestingly, the authors also evaluated the economic costs of CQD‐based catalyst versus Ru catalyst, indicating the overwhelming superiority of CQDs.

Kong et al. developed the CPDs‐decorated ultrathin Bi_2_WO_6_ nanosheets as photocatalyst for CO_2_ methanation, which were prepared by a highly facile hydrothermal approach.^[^ ^121^ ^]^ The upconversion photoluminescence of CPDs allowed this binary nanocomposite to be wide‐spectrum‐responsive, especially in vis–NIR region (Figure 11h). The appropriate spectral coupling between Bi_2_WO_6_ nanosheets and CPDs, the electron‐extraction capability of CPDs, as well as the exposed (001) active facets brought a significant conversion of CO_2_ to CH_4_ with a rate of 7.19 µmol g_cat_^−1^ after 8 h visible illumination. Dai and colleagues proposed a novel photothermal coupled configuration for CO_2_ converting into CH_4_ in virtue of CPDs decorated Cu‐TiO_2_.^[^ ^122^ ^]^ In terms of mechanism, the thermodynamic nonspontaneous process of CO_2_ to CH_4_ was split into a binary spontaneous process including the reduction of CO_2_ to CO first catalyzed by Cu(I), then CO was reduced to CH_4_ by H_2_O (Figure 11i). CPDs here acted as the electron reservoir for storage and transportation of electrons from TiO_2_ excited by UV light, thus further driving the redox cycle of Cu(I)/Cu(II). This creative coupling reaction strategy supplied different consideration orientation to achieve thermodynamic nonspontaneous reactions.

### 5.4 Photocatalytic N_2_ Fixation

Although nitrogen makes up over 78% of the atmosphere, it is hard to be effectively converted and utilized because of the pretty high N≡N bond energy.^[^ ^123^ ^]^ Haber discovered a route for synthesizing ammonia by reacting atmospheric nitrogen with hydrogen in the presence of iron at high pressure and temperature and this Haber–Bosch process (20–40 MPa, 673–873 K) is still used in industrial nitrogen fixation till now.^[^ ^124^ ^]^ Unfortunately, the extreme condition will consume large amounts of fossil fuels and release million tons of greenhouse gases each year. For eco‐friendliness and energy sustainability's sake, moderate methodologies for artificial nitrogen fixation like photocatalysis are hugely needed.

**Figure 12:**
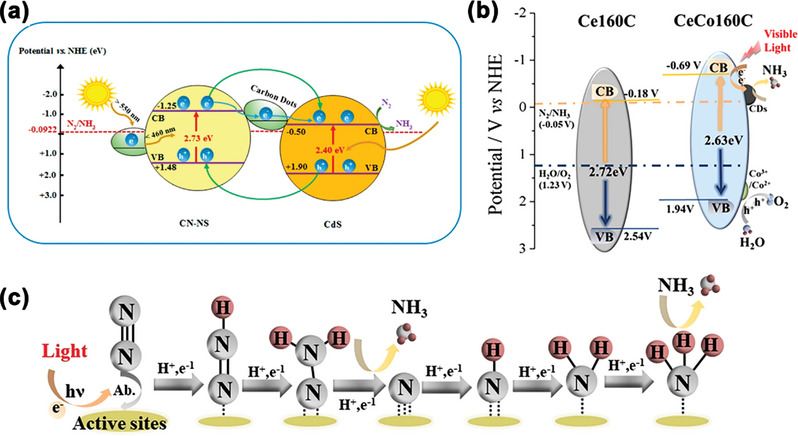
a) Schematic illustration of photocatalytic N_2_ fixation by CN‐NS/CdS. Reproduced with permission.^[^ ^125^ ^]^ Copyright 2019, Elsevier. b) Schematic illustration of photocatalytic N_2_ fixation by CDs/Co double doped CeCO_3_OH. c) Proposed reaction mechanism during photocatalytic N_2_ fixation. Reproduced with permission.^[^ ^127^ ^]^ Copyright 2022, Elsevier.

Among the catalysts applied in photocatalytic nitrogen fixation, metallic semiconductors occupy the majority position. For the positive electron affinity of nitrogen atoms, it is liable to form interactions between the antibonding *π*‐orbitals of N and metal cation sites, which can activate N≡N bonds and accelerate nitrogen fixation.^[^ ^73^ ^]^ CDs as superior electron mediator and light harvester, enable to promote charge separation and broaden light absorption range during photocatalytic process when combining with proper semiconductors. In 2018, Khalilabad et al. introduced CPDs into g‐C_3_N_4_ nanosheets (CN‐NS) decorated with CdS NPs through microwave‐assisted method.^[^ ^125^ ^]^ The upconversion photoluminescence of CPDs helped convert long wavelength light (>550 nm) to short wavelength light which can be absorbed by CN‐NS directly. Besides, CPDs acted as an electron mediator to facilitate the continuous flow of electrons from CN‐NS to CdS NPs (**Figure** **12**a), and further to antibonding orbitals of adsorbed N_2_ molecules. Through the mechanisms above, the optimized ternary catalyst managed an ammonia generation rate of 2157.4 µmol L^−1^ g^−1^ h^−1^ much higher than CdS, CN‐NS or binary catalyst. Likewise, Fei et al. designed Bi_2_WO_6_ nanoflowers modified with GQDs.^[^ ^126^ ^]^ GQDs/Bi_2_WO_6_ nanoflowers composites had better carrier separation, less charge recombination, and smaller resistance than bare Bi_2_WO_6_, leading to an improved nitrogen fixation efficiency.

Cerium dioxide and cerium hydroxycarbonate are considered as promising photocatalysts for nitrogen fixation due to the rich surface defects, good redox performances between Ce(IV) and Ce(III). Recently, Zhang et al. designed CPDs/Co ions double doped CeCO_3_OH, which introduced local active sites and promoted surface absorption in visible range (Figure 12b).^[^ ^127^ ^]^ In photocatalytic N_2_ reduction tests, CPDs/Co ions double doped CeCO_3_OH possessed rates of production up to 1.1 and 0.65 mmol g_cal_^−1^ h^−1^ under full spectrum and visible light, and the catalysts without CPDs exhibited negligible activity. According to FT‐IR spectra, the author speculated the route for N_2_ reduction was likely to follow the associative distal mechanism (Figure 12c). The adsorbed N_2_ molecule is first combined with a proton and a photoinduced electron to form a —N=N—H bond. More protons and electrons would react with —N=N—H step by step, until generating NH_3_. Similarly, this group also developed an all‐solid Z‐scheme heterojunction of CeO_2_‐CPDs‐mixed metal hydroxides (MOH), which CPDs acted as an electron mediator inside.^[^ ^128^ ^]^

### 5.5 Photochemical Synthesis

Green harmless catalytic synthesis is always significant research proposition in history of synthetic organic chemistry. In past decades, the emergence of light‐mediated catalysis endued new vitality to novel synthetic methodologies and bond construction protocols in organic synthesis.^[^ ^129^ ^]^ In virtue of the elevated energy efficiency, atom economy, and sustainability, photochemical synthesis is anticipated to play crucial roles in broader platforms like fine chemical industries or pharmaceutical companies, rather than laboratory research only.^[^ ^130^ ^]^ CDs‐based materials as emerging class of low‐cost photoredox catalyst, have been widely investigated in photoinduced cleavage and construction of carbonous chemical bonds. Here in this section, we will discuss the instances of CDs‐based photoinduced redox reactions involving specific organic synthetic methodology and macromolecular synthesis.

#### 5.5.1 Photochemical Organic Methodology

**Figure 13:**
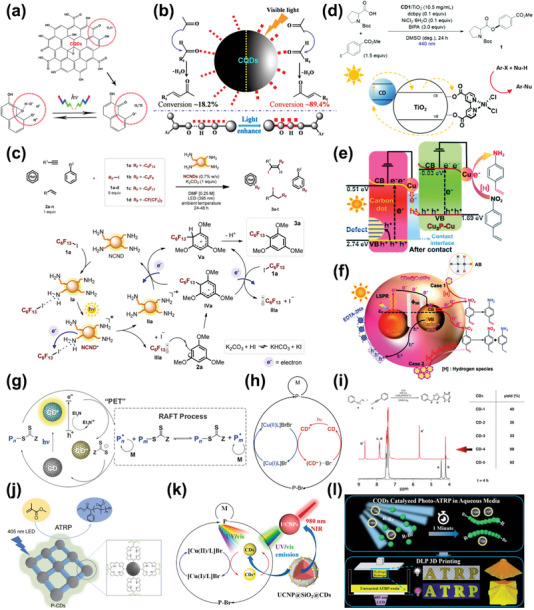
a) The proposed light‐induced proton generation mechanism of CQDs under visible light irradiation. Reproduced with permission.^[^ ^131^ ^]^ Copyright 2014, The Royal Society of Chemistry. b) Schematic illustration of photoenhanced catalytic mechanism of H‐Bond catalysis by CQDs under visible light irradiation. Reproduced with permission.^[^ ^133^ ^]^ Copyright 2014, American Chemical Society. c) Schematic illustration of CDs‐based photocatalytic perfluorohexylation mechanism. Reproduced with permission.^[^ ^134^ ^]^ Copyright 2019, Wiley‐VCH. d) Schematic illustration of CDs‐based metallaphotocatalytic carbon‐heteroatom cross‐couplings. Reproduced with permission.^[^ ^135^ ^]^ Copyright 2021, The Royal Society of Chemistry. (e,f) Schematic illustration of the photocatalytic mechanism of chemoselective hydrogenation of 4‐NS by Cu_3_P/CDs/Cu (e) and CDs/CuNPs (f) under visible light irradiation. (e) Reproduced with permission.^[^ ^136^ ^]^ Copyright 2021, The Royal Society of Chemistry. (f) Reproduced with permission.^[^ ^137^ ^]^ Copyright 2021, The Royal Society of Chemistry. g) Schematic illustration of CDs‐based PET‐RAFT polymerization. Reproduced with permission.^[^ ^139^ ^]^ Copyright 2018, Wiley‐VCH. h) Schematic illustration of CDs‐based PET‐ATRP polymerization. Reproduced with permission.^[^ ^140^ ^]^ Copyright 2020, Wiley‐VCH. i) CDs‐based photoinduced CuAAc reaction. Reproduced with permission.^[^ ^141^ ^]^ Copyright 2021, Wiley‐VCH. j) Schematic illustration of PET‐ATRP polymerization catalyzed by CDs‐based porphyrin covalent network. Reproduced with permission.^[^ ^142^ ^]^ Copyright 2022, Wiley‐VCH. k) Schematic illustration of PET‐ATRP polymerization catalyzed by UCNP@SiO_2_@N‐CDs under full‐spectrum irradiation. Reproduced with permission.^[^ ^143^ ^]^ Copyright 2022, American Chemical Society. l) A minute‐level PET‐ATRP polymerization catalyzed by CQDs in aqueous solution and schematic illustration of DLP 3D printing. Reproduced with permission.^[^ ^144^ ^]^ Copyright 2022, American Chemical Society.

Solid acid catalysts are remarkably preferable to conventional liquid acids in acid‐catalyzed organic synthesis. However, it is hard to design such a solid acid catalyst with strong acid sites and reaction controllability. As reported by Liu and co‐workers, they proposed a type of 5–10 nm CQDs exhibiting strong photoinduced proton generating ability under visible light irradiation.^[^ ^131^ ^]^ CQDs were competitive candidates for acid‐catalyzed reactions due to their highly functionalized nanostructure with light‐controllable proton concentration regulation in aqueous solution. The local CQDs tended to undergo isomerization of the carbonyl groups toward epoxy groups and formed an intermediate that epoxy groups linked with HO‐, thus released protons under illumination (**Figure** **13**a). A series of classical acid‐catalyzed reactions including esterification, Pechmann condensation, and aldol condensation can be efficiently catalyzed by this CQDs with conversions around 40%. On the other hand, CQDs displayed a prominent decrease in photocatalytic activity after treatment by hydrazine hydrate or hydrogen plasma, which further confirms the crucial roles of surface oxygenic functional groups. Similarly, MacFarlane's group also proposed a ring‐opening reaction catalyzed by a sulfonated CQDs as phototriggered proton donor.^[^ ^132^ ^]^ This S‐doped CQDs were generally applied in the ring‐opening of epoxides with methanol or other primary alcohols as nucleophile. The reaction completed in few minutes with conversion up to 98% and excellent selectivity of products.

The presence of secondary bonding interactions between the abundant functional groups on the surface of CDs and the reaction substrate is always extremely beneficial for driving the reaction. Kang et al. reported a photoenhanced Aldol reaction driven by CQDs, prepared by an electrochemical etching method from graphite rod.^[^ ^133^ ^]^ Herein, CQDs were utilized as effective heterogeneous nanocatalysts for Aldol condensation between acetone and substituted aromatic aldehydes. The reaction conversion under light irradiation was several times higher than in dark. The author researched the mechanism by comparing different CQDs with or without hydroxyl groups on surfaces and operated reaction under light irradiation or in dark, respectively. CQDs without surface hydroxyl groups revealed scarcely any reactivity in this Aldol condensation no matter under visible light or not. The photoinduced electrons tended to transfer from O—H···O region to the core of CQDs due to the efficient electron accepting property (Figure 13b). Consequently, the interfacial H‐bond built by hydroxyl groups was strengthened and the carbonyl bond of aldehydes was activated which was prone to nucleophilic reactions. In 2019, Filippini and Prato came up with a photochemical radical perfluoroalkylation strategy for arene and olefin derivatives.^[^ ^134^ ^]^ The amino rich N‐CPDs utilized as photocatalyst were prepared using arginine and EDA as precursors through microwave‐assisted hydrothermal method. These N‐CPDs showed strong absorption in UV region with a band tail to 500 nm. During the reaction process, the amino groups on N‐CPDs would primarily form halogen bond adduct with substrate molecules, thereby promoted the photoactivation of perfluoroalkyl iodides. Then the N‐CPDs were excited by 395 nm light irradiation, N‐CPDs with low reduction potential readily photoinduced a single‐electron transfer to perfluoroalkyl iodides. Accordingly, the formed electrophilic radicals reacted with electron‐rich reactant, followed by further oxidation and deprotonation to yield perfluorinated compounds (Figure 13c).

Besides the examples of single CDs photocatalysis mentioned above, CDs can also hybrid with heterogeneous semiconductors or metals NPs to enhance absorption and avoid the rapid recombination of photoinduced carriers. Pieber's and Delbianco's groups reported a photocatalytic carbon‐heteroatom cross‐couplings reaction, which took carbohydrate‐based CPDs, which were immobilized on TiO_2_ in combination with nickel complexes, as photocatalyst (Figure 13d).^[^ ^135^ ^]^ The composite exhibited a superior absorption in visible region comparing with TiO_2_. The author performed plenty of photocatalytic cross‐coupling reactions between aryl halides and alcohol, thiol, sodium sulfonate or sulfonamide, constructing C—O, C—S or C—N bonds with satisfactory yields. In addition, Hu et al. gave two different CPDs‐based photocatalytic selective hydrogenation strategies of 4‐nitrostyrene (4‐NS) to 4‐aminostyrene (4‐AS) with ≈100% conversion and more than 99% selectivity in only few minutes. One of the multicomponent photocatalysts were composed of Cu_3_P‐CPDs‐Cu obtained by pyrolysis.^[^ ^136^ ^]^ This Z‐scheme catalytic system would transfer the excited electrons produced from CPDs to combine with the holes in Cu_3_P, thus avoided holes to attack the vinyl in 4‐NS without sacrificial agent (Figure 13e). In this way, the excited electrons in Cu_3_P, facilitated by surface metallic Cu NPs, were capable for efficient and highly selective photocatalytic reduction of nitro with amino borane (AB). Another route for the same goal was achieved by combining CPDs and Cu NPs.^[^ ^137^ ^]^ The localized surface plasmon resonance effect of CuNPs enhanced the absorption of the nanocomposite and the existence of Schottky barrier at the interface hindered excited electrons back to CuNPs, which extended the lifetimes of photoinduced carriers (Figure 13f). As a result, the nitro of 4‐NS was reduced by the energetic electrons with surface hydrogen species released from AB under visible light irradiation.

#### 5.5.2 Photoinduced Electron Transfer Macromolecular Synthesis

Photochemical synthesis supplied broader horizons and directions for developing contemporary polymeric synthetic chemistry, and such photoinduced electron transfer process has been successfully applied in fields including initiation, molecular modification, regulation of polymeric kinetics and structures.^[^ ^138^ ^]^ In the past years, numerous achievements regarding CDs‐based photocatalytic reversible deactivation radical polymerization (RDRP) have been published, represented by reversible addition–fragmentation chain transfer (RAFT) polymerization, atom transfer radical polymerization (ATRP), etc.

The first employment of CDs in photocatalytic RDRP was reported by Matyjaszewski and co‐workers in 2018 (Figure 13g).^[^ ^139^ ^]^ They fabricated a series of heteroatom‐doped CPDs through hydrothermal method and performed photoinduced electron transfer RAFT (PET‐RAFT) polymerization under the irradiation blue LED (465 nm, 4 mW cm^−2^). Sulfur‐doped CPDs showed highest catalytic efficiency to catalyze polymerization of methyl methacrylate (MMA) in DMSO, which can obtain polymer with predictable molecular weight and low polymer dispersity index (PDI) ≈1.1. During the irradiation period, chain transfer agents were reduced by photoinduced electron from excited S‐CPDs, then initiated polymerization to form a dynamic equilibrium between dormant species and the propagating radical chains.

Strehmel and co‐workers further researched CDs as photosensitizer in photocatalytic ATRP.^[^ ^140^ ^]^ CuBr_2_ and alkyl halide were added as catalyst and initiator for ATRP process of MMA under 405 nm light. Photoinduced electron transferred from excited CPDs to [Cu(II)L]Br_2_ generating the corresponding reduction product [Cu(I)L]Br, then initiating the living radical polymerization by reaction with alkyl halide (Figure 13h). However, the product polymer's PDI resided ≈1.5 revealing the existence of chain termination. In the following investigation, they expanded the variety of biomass‐based CPDs and explored their applications as photosensitizers in photocatalytic free radical polymerization, ATRP and photoinduced CuAAC reactions under visible light (Figure 13i).^[^ ^141^ ^]^ In their another work, a CPDs‐based porphyrin covalent network was synthesized through Alder‐Longo reaction (Figure 13j).^[^ ^142^ ^]^ The enhanced electrochemical and photonic properties of such heterogeneous material enabled a facilitation of photo‐ATRP.

Broadband range light, especially NIR light mediated polymerization has received widespread attention due to the promising potential in solar‐catalytic polymerization and biochemical applications. Pang et al. proposed a strategy of combining pyridine nitrogen doped CPDs with upconversion nanoparticles (UCNPs) to realize photocatalytic ATRP in the full spectrum (Figure 13k).^[^ ^143^ ^]^ The well‐controlled photo‐ATRP process can be achieved from UV/vis light to NIR even with an extremely low loading of CuBr_2_/ligand and UCNP@SiO_2_@N‐CPDs composite catalyst. Later on, the author first introduced CPDs as photocatalyst into aqueous solution and developed a minute‐level photo‐ATRP.^[^ ^144^ ^]^ Such extraordinary performance of photo‐ATRP was mainly attributed to a higher equilibrium constant *K*_ATRP_ in aqueous environment and high dielectric constant of water, which allowed for stable and rapid propagation of reactive radicals. This ultrafast polymerization process was subsequently applied to additive manufacturing for microscale DLP 3D printing (Figure 13l).

Except for the photocatalytic RDRP mentioned, CDs were also utilized to synthesize conductive polymers (polyaniline, polypyrrole), polypeptides, and proteins.^[^ ^145^ ^]^ In the photocatalytic formation of polypeptides, the existence of oxygen containing groups allowed CQDs to aggregate amino acids on their surfaces. Hydroxyl groups endowed CQDs with strong photoinduced acidity, which was responsible for the catalytic formation of peptide bonds under visible light irradiation. The peptide would be oxidized by photogenerated holes ulteriorly, thus to form the tertiary structure of proteins.

## 6 Conclusion and Future Outlook

Here in this review, we highlighted recent advances of CDs‐based photoinduced chemical reactions, including the structural features, synthesis methods, photoelectric properties of CDs and CDs‐based photocatalytic applications. The unique nanoscale structure and integrated graphitized sp^2^ domains inside endowed CDs superior optical and photoelectrical properties. In addition, diverse fabrication methods offered variety of tools for CDs preparation or proceeded further modification. The nature of UCPL and photoinduced charge migration capability make CDs efficient wide‐response photocatalyst. Furthermore, by focusing on performance and mechanism, photoinduced reactions including hydrogen generation, pollutant degradation, CO_2_ conversion, N_2_ fixation, and photochemical organic synthesis based on individual CDs and CDs‐based composite were systematically discussed in detail. In view of the future designs of CDs‐based photocatalysts, it is imperative to have an in‐depth discussion on the structure of CDs and corresponding structure–activity relationship in photocatalytic systems as different roles, such as photosensitizer, photocatalyst or combining with other semiconductors as cocatalyst. For instance, adjusting structural natures such as increasing the degree of graphitization of carbon cores, enables to enhance the stability of CDs. If high proportion of molecular states exists in CDs, it is prone to undergo photobleaching under photoexcitation, resulting in CDs‐based catalyst deactivation. Moreover, higher graphitization extent of also brings superior charge migration ability for CDs. On the other hand, plentiful functional groups located on the surface of CDs can form some interactions with specific substrates, thus to lower the energy barrier and facilitate the catalytic reaction proceeding of CDs as photocatalyst. Noteworthily, these functional groups are also beneficial for CDs as cocatalyst combing with other semiconductors.

Although great efforts have been devoted to CDs research and obtained achievements in photocatalytic applications, some major issues still remain for further investigation.

1. In CDs‐based photoinduced reactions, absorption, charge migration ability, bandgap, solubility, and some other properties of CDs are strongly associated with the performance of photocatalysis. These properties influencing the catalytic performance should be studied in depth to propose universal laws. As a consequence, besides utilizing the common steady state or transient spectra, introducing advanced in situ photoelectrical characterizing techniques (transient photoinduced current, transient photoinduced voltage, etc.) is imperative and beneficial for monitoring the electron transfer mechanisms and interfacial electron transport kinetics, thus to figure out the acting sites and in situ catalytic reaction kinetics in dynamic CDs‐based photocatalytic process with multi‐timescale.^[^ ^10a^ ^]^
2. For CDs‐based photocatalytic systems, long‐term chemical stability and photostability are the top priorities. Besides the internal stability enhancement of CDs mentioned before by increasing the degree of graphitization, avoiding photocorrosion is also quite essential. Therefore, the accumulated photoinduced electrons or holes must be consumed in time. For this, common strategy is the addition of the corresponding sacrificial agent to the catalytic system. Inspired by the overall water splitting, how to design delicate catalytic systems that utilize both electrons and holes efficiently and rationally to achieve multireaction orthogonal catalytic processes without the addition of sacrificial agents should be an interesting proposition in the future.
3. Novel peculiarities of CDs applied in photocatalysis require further exploration. For instance, the chirality of CDs as a new developed property mainly applied in chiral drug recognition or bulk chiral materials fabrication. In this review, we have introduced examples of CDs as photoredox catalysts for organic transformations. If the chiral CDs are capable for photocatalytic organic synthesis, substrate molecule may connect with chiral part located on CDs to form asymmetric intermediates under photoexcitation, thus possible to realize one‐step photocatalytic asymmetric organic synthesis based on CDs. Moreover, this conception will not be limited to stereocontrolled transformations of small molecules, but even promising for the synthesis of macromolecules with stereoregularity.
4. The ultimate goal of scientific research is to focus on life‐oriented applications, so if we intend to popularize CDs‐based photocatalysis in the future, the cost effectiveness of production capacity and the environmental impacts are key considerations. However, few reports have been published to evaluate the performance of CDs‐based catalyst in large‐scale photocatalytic applications. To the best of our knowledge, we suppose that photochemical synthesis is the most promising to be widely utilized. Besides the facile procedure, low cost and high efficiency, CDs‐based photochemical synthesis will also face less difficulties about equipment in terms of industrial scale‐up.

In summary, despite CDs still have deficiencies awaiting exploitation, the merits of CDs applied in photocatalysis over other semiconductor nanomaterials are prominent. Therefore, for the sake of producing CDs‐based materials in high quality and good uniformity on large scale, it is necessary to develop controllable CDs synthesis or even atomic precise synthesis with normative purification procedure based on different features of CDs, and this will beneficial for investigating structure–functional relationship as well. Additionally, as the increasing computing power and latest developed models of machine learning, it will provide stronger technical support for guiding the design and synthesis of CDs‐based photocatalysts. I hope the discussion in this review and aforementioned issues can provide novel enlightenment for CDs‐based photoinduced chemical reactions in the future.

## Conflict of Interest

The authors declare no conflict of interest.
